# Crosstalk between innate immune signaling pathways and integrated TLR, NLRP3 inflammasome, cGAS–STING, and NF-κB networks in sepsis

**DOI:** 10.3389/fcell.2026.1935200

**Published:** 2026-09-11

**Authors:** Xiaoxi Du, Han Qiao

**Affiliations:** 1 The Second Clinical College of China Medical University (Shengjing Hospital), Shenyang, China; 2 Department of Critical Care Medicine, The First Hospital of China Medical University, Shenyang, China

**Keywords:** cGAS-STING, innate immunity, NF-κB, NLRP3 inflammasome, sepsis, signaling pathway, TLR

## Abstract

Sepsis is a life-threatening syndrome characterized by a dysregulated host response to infection that culminates in systemic inflammation, immune dysfunction, and multiple organ failure. Despite advances in supportive care, the molecular mechanisms underlying sepsis remain incompletely understood, largely due to the complex interplay among innate immune signaling pathways rather than the activation of individual pathways in isolation. Recent evidence indicates that extensive crosstalk between toll-like receptor (TLR), NLRP3 inflammasome, cyclic GMP–AMP synthase-stimulator of interferon genes (cGAS–STING), and nuclear factor-kappa B (NF-κB) signaling networks orchestrates the instigation, amplification, and resolution of inflammatory responses during sepsis. These interconnected pathways collectively regulate pathogen recognition, cytokine production, inflammasome activation, type I interferon signaling, pyroptosis, and immune cell reprogramming, while simultaneously interacting with metabolic, oxidative stress, autophagic, and programmed cell death pathways. This review comprehensively examines the molecular architecture and dynamic interactions among these signaling networks, highlighting the shared adaptor proteins, regulatory feedback loops, and signaling hubs that determine the transition from protective host defense to uncontrolled hyperinflammation and subsequent immunosuppression. We further discuss how mitochondrial dysfunction, reactive oxygen species (ROS), damage-associated molecular patterns (DAMPs), epigenetic modifications, and non-coding RNAs (ncRNAs) modulate these signaling circuits and contribute to organ-specific injury in sepsis. Emerging evidence from single-cell transcriptomics, spatial multi-omics, and systems biology methods is also explored to provide a network-level understanding of immune dysregulation and identify novel biomarkers and therapeutic targets. Finally, we summarize current and emerging therapeutic strategies aimed at modulating innate immune signaling, including inhibitors of TLRs, NLRP3 inflammasome, STING, and NF-κB, as well as combination and precision medicine approaches. By integrating canonical and emerging signaling pathways into a unified molecular framework, this review provides new insights into the pathogenesis of sepsis and highlights promising directions for the advancement of targeted immunomodulatory therapies capable of cultivating clinical outcomes.

## 1 Introduction

**FIGURE 1:**
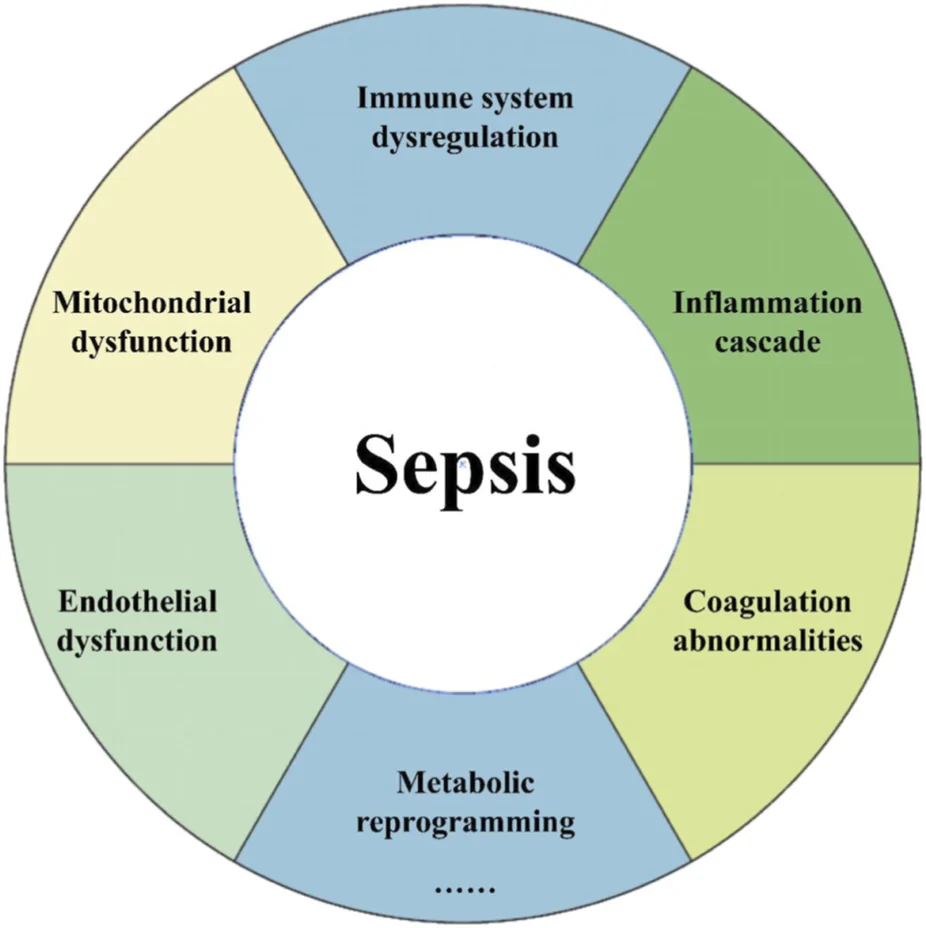
Schematic overview of the pathological mechanisms of sepsis. The diagram illustrates the sequential activation of innate immune signaling following pathogen recognition, including PRR engagement, cytokine storm, immunosuppression, endothelial dysfunction, coagulopathy, and progression to multiple organ dysfunction syndrome (MODS). Key cellular players and molecular mediators are indicated (Xian et al., 2025). Reprinted from Front. Immunol., 16:1679493 Di X et al., “ Metabolic reprogramming tailors T cell immunity in sepsis “, Copyright (2026), with permission from Frontiers.

Sepsis is a life-threatening syndrome characterized by host response dysregulation to infection and acute organ failure and remains among the most prevalent causes of mortality in intensive care settings globally (Saavedra-Torres et al., 2025). Despite advances in antimicrobial therapies and critical care medicine, it remains a burdensome syndrome because of its complex pathophysiology and the lack of effective, targeted therapies (Kox et al., 2026). Compared to other infections, the systemic nature of sepsis warrants a response characterized by immune dysregulation, altered metabolism, organ failure, and multiple organ dysfunction syndrome (MODS) (Wiersinga and van der Poll, 2026). The challenges posed by the constantly evolving and diverse nature of the immune response and the clinical presentation of the syndrome fail to provide the impetus to develop effective therapies that will benefit the majority of patients (Chowdhury et al., 2026). There is an imperative need to better define and comprehend the immune dysregulation in response to infection and the various host and pathogen interactions (Figure 1) (Ovali and Percin, 2025; Xian et al., 2025).

The innate immune system acts both as the first line of defense against invading pathogens and as the system responsible for the initiation of the subsequent adaptive immune responses (Kumar et al., 2024; Zhang Y. et al., 2026). The immune cells such as macrophages, monocytes, and neutrophils, as well as dendritic cells and resident sentinel cells, use pattern recognition receptors (PRRs) to rapidly detect invading pathogens (Saavedra-Torres et al., 2025). These receptors bind to pathogen-associated molecular patterns (PAMPs) as well as damage-associated molecular patterns (DAMPs) and respond, in a germline-encoded and invariant manner, to diverse infecting microbes such as bacteria, viruses, fungi, and parasites (Yilmaz et al., 2026). The activation of a PRR or several PRRs initiates a coordinated response, including the generation of cytokines and chemokines, antimicrobial responses, and recruitment and repair responses (Liu and Liang, 2025). During homeostasis, the immune system maintains a balance amongst its diverse responses and dampens injury to host tissues. This balance is lost in sepsis, when unregulated PRR activation and high levels of inflammation lead to multiple organ failure (Kox et al., 2026). Among the diverse PRRs, Toll-like receptors (TLRs), nucleotide-binding oligomerization domain (NOD)-like receptors (NLRs), and cytosolic DNA sensors are some of the most important PRRs that modulate innate immune system activation during sepsis (Wiersinga and van der Poll, 2026; Lee et al., 2023). TLRs identify microbial ligands in either the extracellular space or in endosomes. TLRs then signal through myeloid differentiation primary response protein 88 (MyD88) and Toll/interleukin-1 receptor-domain-containing adaptor-inducing interferon-β (TRIF), two adaptor proteins, and activate downstream transcription factors, nuclear factor-kappa B (NF-κB), activator protein-1 (AP-1), and the interferon regulatory factors (IRFs) (Qian C. et al., 2026). Pro-inflammatory cytokines and type I interferon are then produced and participate in anti-microbial immunity. Pyroptosis and the maturation of interleukin-1β (IL-1β) and interleukin-18 (IL-18) are the result of caspase-1 activation and are characteristic of the NLRP3 inflammasome, which is also a platform for the integration of intracellular signals of danger (Heinke, 2025; Zheng et al., 2021). Additionally, the cyclic GMP-AMP synthase-stimulator of interferon genes (cGAS-STING) pathway has emerged as a key player in innate immunity that connects cytosolic DNA sensing, inflammation, and mitochondrial dysfunction. cGAS-STING modulates type I interferon responses in both infectious and non-infectious inflammatory conditions (Hooftman et al., 2026).

Although there has been a tendency to study these signaling pathways separately, evidence suggests that these pathways are highly interrelated signaling networks (Saavedra-Torres et al., 2025). The TLR, NLRP3 (NOD-, LRR- and pyrin domain-containing protein 3) inflammasome, cGAS-STING, and NF-κB pathways all interact to allow immune cells to respond to a combination of harmful microorganisms and triggers from within the body (Kox et al., 2026). For instance, the NF-κB pathway, activated by TLRs, serves as the initial signal to construct the NLRP3 inflammasome, as it also promotes the transcription of inflammasome components and pro-inflammatory cytokines (Qian C. et al., 2026). NLRP3, on the other hand, enhances NF-κB-mediated inflammation by the release of IL-1β and IL-18, establishing a positive feedback mechanism for the cytokine storm seen in severe sepsis. Likewise, stimulation of the cGAS-STING pathway by bacterial or mitochondrial DNA also results in the transcription of NF-κB target genes and the activation of pathways associated with interferon regulatory factor 3 (IRF3), inflammasome, autophagy, and regulated cell death (Hooftman et al., 2026). These effects promote a strongly interconnected signaling network that determines the intensity and duration of the body’s immune response. Due to its role as a master transcriptional regulator, NF-κB connects many upstream pathways of the innate immune system to downstream pathways of the immune responses to inflammation (Zhong et al., 2016). NF-κB activation drives the transcription of hundreds of target genes. These include genes encoding cytokines, chemokines, antimicrobial peptides, apoptotic regulators, and immune cell survival factors. (Chen Y. et al., 2025). However, persistent or excessive activation of NF-κB leads to the formation of an excessive amount of inflammatory mediators, the disruption of the endothelial barrier, the loss of normal function of mitochondria and other systems, and injury to various body tissues, all of which contribute to the advancement of septic shock (Yilmaz et al., 2026). The protective and pathological functions of NF-κB demonstrate the importance of the precise signaling pathways that must be maintained throughout the various stages of sepsis (Liu et al., 2025).

Several studies have demonstrated that many intracellular pathways regulate innate immune system signaling. Mitochondrial dysfunction, excessive production of reactive oxygen species (ROS), stress in the endoplasmic reticulum (ER), dysregulated calcium, and autophagy impairment also affect the activation of NF-κB, cGAS–STING, and various other receptors (Han et al., 2024). Nonetheless, some epigenetic alterations and stress in the immune metabolism system have also impacted the regulation of innate immune system cells and the state of sepsis (Wu D. et al., 2023). A key characteristic of sepsis is a shift from an initial hyperinflammatory state to a prolonged phase of immune dysfunction, or immunoparalysis. The early stages of sepsis are marked by the activation of the innate immune response to clear infection (Ovali and Percin, 2025). However, a sustained inflammatory response results in damage to the endothelium and organs, vascular leakage, and intravascular coagulopathy. This is then accompanied by severe immunosuppression, including lymphocyte death, loss of antigen-presenting capacity, T-cell exhaustion, and increased myeloid-derived suppressor cells, along with a heightened risk of opportunistic infections (Gao X. et al., 2025). The transition reflects the remodelling of immune signaling networks and illustrates the body’s inability to restore immune homeostasis (Ovali and Percin, 2025).

High-performance research technologies help model complex systems (Wiersinga and van der Poll, 2026). High-dimensional bulk and single-cell molecular profiling, such as transcriptomics, proteomics, and metabolomics, can help us define the immune regulation and cellular interactions associated with sepsis (Isac et al., 2024). The application of these technologies may allow us to characterize signalling networks, identify novel biomarkers, and create new regulatory circuits to assist in stratifying sepsis patients. The integration of high-dimensional data, coupled with the prediction of pathway interactions, provides the opportunity to identify innovative therapeutic approaches that may signal the end of the paralysis of immune homeostasis (Maestraggi et al., 2017). Addressing the molecular interactions of the TLR, NLRP3 Inflammasome, cGAS–STING, and NF-κB pathways will not only advance our understanding of the mechanisms of sepsis and guide the progress of more effective treatment options but will also account for the dismal results of previous anti-inflammatory treatment modalities (Saavedra-Torres et al., 2025). The treatment failures are due to the targeting of singular cytokines and/or signaling components, thus ignoring the entire regulatory framework (Qian C. et al., 2026).

Several prior reviews have examined components of innate immune signaling in sepsis in isolation, TLR-mediated inflammation, NLRP3 inflammasome biology, cGAS-STING in sterile immunity, NF-κB as a therapeutic target and general sepsis immunopathology. While each provides valuable mechanistic insight, none systematically examines the integrated crosstalk among all four pathways simultaneously. The present review fills this gap by providing the first comprehensive molecular framework for TLR–NLRP3–cGAS-STING–NF-κB network interactions in sepsis, with explicit emphasis on shared adaptor proteins, regulatory feedback loops, and multi-target therapeutic strategies.

This review, for the first time, describes the combined interactions of the TLR, NLRP3 inflammasome, cGAS-STING, and NF-κB pathways in sepsis (Saavedra-Torres et al., 2025). We focus on the mechanisms that control activation and reciprocal regulation of these pathways, feedback signaling and their interaction with other pathways involved in mitochondrial function, oxidative stress, autophagy, immunometabolism, and regulated cell death (Kumar et al., 2024; Fitzpatrick, 2019). We also discuss the impact of recent advances in multi-omics and systems immunology on our understanding of innate immune signaling (van der Poll et al., 2021). Finally, we describe new targeting therapies for these interlinked pathways and discuss the new directions that precision immunomodulation can take to improve the clinical outcome of patients with sepsis (Hossan et al., 2026; Mantzarlis et al., 2017).

## 2 Pathophysiology of sepsis

### 2.1 Recognition of pathogens and host-derived signals

Sepsis is thought to commence when an invading organism breaches the defensive barriers of the host and is first sensed by the innate immune system (van der Poll et al., 2021). The detection of invading organisms and the danger signals associated with tissue damage are mainly the responsibility of PRRs (Soares et al., 2023). A host of immune cells, including macrophages, monocytes, neutrophils, dendritic cells, and even endothelial and epithelial cells, possess PRRs (Yilmaz et al., 2026). There are two main classes of immunostimulatory molecules that PRRs are involved in detecting: PAMPs and DAMPs (Qian C. et al., 2026; Gong et al., 2020). The binding of PAMPs and DAMPs to their respective PRRs results in the commencement of intracellular pathways that stimulate both the defense and the inflammatory response (Denning et al., 2019). PAMPs are molecular structures that are similar across species and are found in bacteria, fungi, viruses, and parasites, but are not found in mammalian cells (Wu H. et al., 2025). Examples of PAMPs are lipopolysaccharide (LPS) found in Gram-negative bacteria, peptidoglycan and lipoteichoic acid in Gram-positive bacteria, flagellin, unmethylated CpG, dsRNA, viral ssRNA, and β-glucans and mannans in fungi (Wu H. et al., 2025). These structures interact with various PRRs, which include TLRs, NLRs, CLRs, RLRs, and various cytoplasmic DNA sensors like cGAS (Soares et al., 2023). PRRs initiate downstream signaling pathways when they interact with PAMPs. These pathways are mediated by various adaptor proteins, like MyD88, TRIF, and TRAFs, and also by TAK1 and IKK (Qian C. et al., 2026). These pathways activate NF-κB, IRFs, and MAPKs. These activated factors promote the expression of many inflammatory cytokines and chemokines, adhesion and antimicrobial peptides, as well as type I interferons (McNab et al., 2015). These processes are essential to clear infections, but sustained activation leads to excessive inflammation and injury to tissues. The progression of an infection results in the release of endogenous mediators termed DAMPs/alarmins due to the combination of host cell damage and increased cellular stress (Liu and Liang, 2025). In contrast to PAMPs, DAMPs are released by damaged and dying host cells and cause continued inflammation in the absence of microorganisms (Cicchinelli et al., 2024). Significant DAMPs are HMGB1, ATP, mtDNA, histones, HSPs, crystallized uric acid, S100 proteins, oxidized phospholipids and mitochondrial peptides (Ovali and Percin, 2025). DAMPs and PAMPs share many of the same receptors, including TLR2, 4, 9, the NLRP3 inflammasome, and the cGAS–STING pathway (Ma et al., 2024). Since mtDNA have a bacterial ancestry and is thus a strong ligand for TLR9 and cGAS, the release of DAMPs vitally contributes to the amplification of inflammation and cellular injury, even in the absence of pathogens (Saavedra-Torres et al., 2025).

### 2.2 Hyperinflammation and immunosuppression

The initial phase of sepsis features a hyperactive inflammatory response to pathogenic invasion (Li Y. et al., 2025). PRR activation prompts macrophages, neutrophils, dendritic and endothelial cells to abundantly secrete pro-inflammatory factors such as tumor necrosis factor-α (TNF-α) and interleukins (IL) 1, 6, 18 and 5, and several chemokines (Chen R. et al., 2025). These factors recruit circulating leukocytes to infection sites and increase endothelial permeability and immune response activity (Leick et al., 2014). NF-κB signaling is essential to this process as it controls the transcription of a multitude of inflammatory response genes triggered by TLRs and other PRRs. Simultaneously, the NLRP3 inflammasome is primed, and IL-1β and IL-18 are processed, along with the induction of a highly inflammatory death process, pyroptosis, which causes the necrotic release of DAMPs (Ustundag, 2026). The cGAS–STING pathway, in conjunction with NF-κB and inflammasome activation, also generates an inflammatory response directed by type I interferons (Han et al., 2024). The removal of microbes is the intended purpose of this rapidly activated and interconnected signaling response, which is all too often excessive and dangerous (Hooftman et al., 2026). Complement activation, NETs, and other inflammatory and coagulation responses lead to organ dysfunction and progressive damage through thrombotic hypoxia (Markiewski et al., 2008; Mrozewski et al., 2024).

The prevailing theory posited a linear model of hyperinflammation followed by a relative state of immunosuppression, and the evidence from the last few decades challenges this model (Prajapati et al., 2026). Several studies have revealed the existence of pro- and anti-inflammatory processes from the early phases of sepsis and throughout the course of the syndrome (Wiersinga and van der Poll, 2026). The hyper-inflammatory response and uncontrolled pro-inflammatory response lead to compensatory sepsis-induced immunosuppression and a state of immunoparalysis in which patients are more likely to develop secondary infections of poor prognosis (Zhou et al., 2026). Immunosuppression is characterized by the extensive apoptosis of T lymphocytes (CD4 and CD8), dendritic cells, and B cells, and the impairment of antigen presentation and expression of major histocompatibility complex II (MHC II) molecules by the expanding population of regulatory T cells and myeloid-derived suppressor cells (Sabins et al., 2016; Padovani and Yin, 2024). Monocyte HLA-DR expression is diminished, and T cells are functionally exhausted and express checkpoint molecules (PD-1, PD-L1, TIM-3, and LAG-3) (Sabins et al., 2016; Yan Z. et al., 2025). The persistent presence of IL-10 and transforming growth factor-β (TGF-β) leads to a state of immune suppression by blocking antigen presentation and provoking a state of endotoxin tolerance (Fu et al., 2023). There is also the phenomenon of immunometabolic reprogramming and changes at the epigenetic level, which combine to result in a depressed state of endotoxin tolerance (Carson et al., 2011). As a consequence, the ability to combat infections is diminished, leading to persistent infections and an extended stay in the intensive care unit (Zhao et al., 2023).

### 2.3 Organ dysfunction and organ failure

The hallmark of sepsis is the formation of acute dysfunction of one or more organs. This is a result of the interplay of many factors, including inflammatory mediators, injuries to the endothelium, alterations in the coagulation process, a failure in metabolism, dysregulation of the immune response, and mitochondrial dysfunction (He et al., 2024). Not all organ failure is a result of direct infection by microorganisms (Kharga et al., 2023). Organ failure is primarily the result of an unbalanced and exaggerated response of the host (Gunst et al., 2013). Endothelial activation and injury cause an inflammatory response, which promotes an increased permeability of blood vessels, the destruction of the endothelial glycocalyx, the adhesion of leukocytes to the endothelium, the activation of the coagulation cascade, disseminated intravascular coagulation (DIC) and microvascular thrombi (Wiersinga and van der Poll, 2026). Thrombi cause the flow of blood through the microcirculation to be inadequate, which, along with the maintenance of the hemodynamic status at the systemic level, contributes to the failure of the organs to be adequately supplied with oxygen and to be functionally normal (Shirakabe et al., 2023). Injured mitochondria cause organ failure by creating an inflammatory response and failing to produce adequate cellular energy (ATP) and ROS (Hu D. et al., 2024). Energized mitochondria cause the plasma membrane to become permeable to mitochondrial DAMPs and initiate the inflammasome and STING pathway (Gong et al., 2020). A shift in metabolism also occurs, and, unlike other organ systems, the central nervous system (CNS) and the hepatic system, sepsis causes an acute and rapid failure of the immune system and the detoxification processes of the body (Hu et al., 2023). These mechanisms encompass all the organs and will result in MODS, which is the principal cause of death in severe sepsis and septic shock patients (Maestraggi et al., 2017). Of note, the transition from infection to organ failure is characterized by extensive molecular interactions within innate immune signaling pathways, underscoring the importance of considering the system as a whole when designing therapeutic strategies to target network-level dysregulation rather than specific inflammatory mediators (Nedel et al., 2023).

## 3 Innate immune signaling networks

### 3.1 Pattern recognition receptors

The first responders of the immune system utilize PRRs to identify PAMPs and DAMPs (Chen R. et al., 2025). The indiscriminate localization of PRRs to the plasma membrane, endosomes, and the cytosol allows the immune system to detect pathogens and their effects on the body, regardless of whether they are inside or outside the cell (Yilmaz et al., 2026). The PRRs are responsible for triggering intracellular pathways to produce the inflammatory cytokine and chemokine response, interferon, antimicrobial peptides, and co-stimulatory molecules to eliminate the pathogen (Saavedra-Torres et al., 2025). Pattern recognition receptors are categorized to several major families based on the molecular patterns they recognize and the signaling pathways they mobilize, which are mostly complementary (Table 1) (Liu and Liang, 2025).

**TABLE 1:** A summary of the various types of pattern recognition receptors (PRRs) (Chen R. et al., 2025; Takeuchi and Akira, 2010).

No.	Class	Representative receptors
1	TLRs	TLR1-TLR10 (in humans), localized in the plasma membrane and endolysosomes, the origin of ligands is from bacteria and viruses
2	CLRs	MINCLE, DNGR1 (or CLEC9A), CLEC8A CLEC12A, CLEC7A (or Dectin-1), localized in the plasma membrane, origin of ligands is from fungi
3	NLRs	NLRA, NLRB, NLRC, NLRP, and NLRX1, localized in the cytoplasm, origin of ligands is from bacteria
4	RLRs	LGP2, MDA5, and RIG-I, localized in the cytoplasm, origin of ligands is from RNA viruses
5	Cytoplasmic DNA sensors	cGAS and AIM2
6	Extracellular soluble PRMs	Pentraxin (CRP, APCS, and PTX3), collectin, and ficolin
7	iPRRs	CD300a/f, Siglec-2, 3, and 5–11, CEACAM1 LILRB1, LILRB3, TIGIT, PVR, LAIR-1, and SIRL-1

Abbreviations: AIM2 absent in melanoma 2, APCS, amyloid P component serum, CRP C-reactive protein, CLEC9A C-type lectin domain containing 9A, CytC cytochrome C, CEACAM1 carcinoembryonic antigen cell adhesion molecule 1, DNGR1 dendritic cell natural killer lectin group receptor 1, iPRRs, inhibitory PRRs, LGP2 laboratory of genetics and physiology 2, LILRB1 leukocyte immunoglobulin-like receptor subfamily B member 1, LAIR-1, leukocyteassociated immunoglobulin-like receptor 1; MINCLE, macrophage-inducible C-type lectin, MDA5 melanoma differentiation factor 5, PTX3 pentraxin 3, SIRL-1, signal inhibitory receptor on leukocytes 1, TIGIT T-cell immunoreceptor with Ig and ITIM, domains.

#### 3.1.1 Toll-like receptors

**FIGURE 2:**
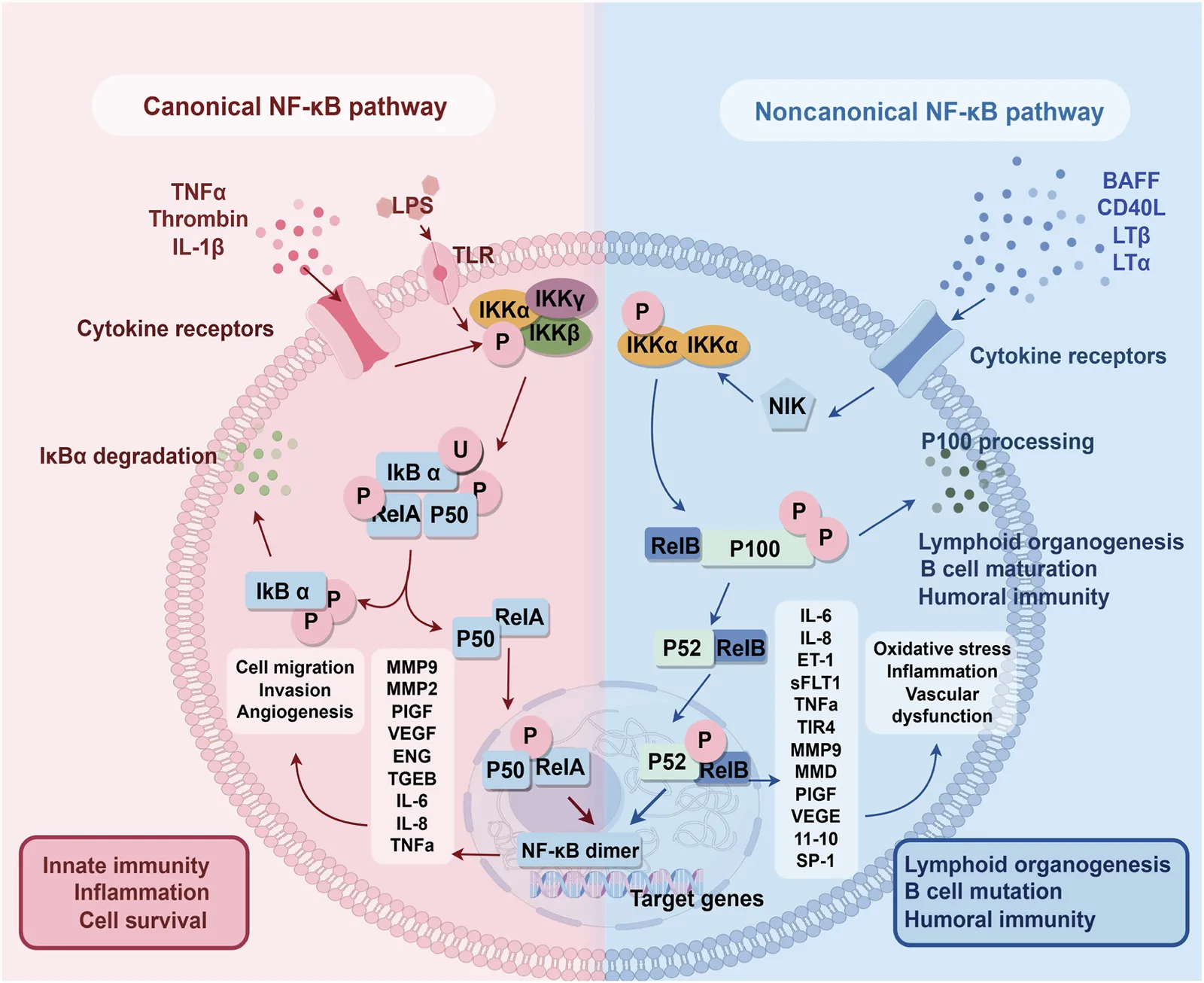
Classical and nonclassical NF-κB signaling in sepsis pathogenesis. In the classical NF-κB pathway, LPS initiate a downstream signaling ending in the activation and nuclear translocation of NF-κB, mediating the expression of inflammatory cytokines to systemic inflammation. NF-κB also regulates apoptosis and immune cell activation, vital in the progression of sepsis. The nonclassical pathway links activation pathways in specific cell types or stimuli, via a variety and blend of kinases, cofactors, and NF-κB subunits, as explained in the diagram. Reprinted from Research, 1; 8:0,811, Tang L et al., “ Decoding Sepsis: Unraveling Key Signaling Pathways for Targeted Therapies”, Copyright (2025), with permission from SPJ.

TLRs are the most studied PRR family and are essential for the first stages of the immune response to pathogen-associated stress and inflammation. The ten TLRs (TLR1-TLR10) identified in humans can recognize and bind to numerous pathogenic ligands (Zheng et al., 2024). The TLRs occur on the cell membrane (TLR1, TLR2, TLR4, TLR5, TLR6, and TLR10) are primarily activated by ligands of bacterial and fungal origin, like lipopolysaccharides and flagellin (Duan et al., 2022). The TLRs found in the endosomes (TLR3, TLR7, TLR8, and TLR9) recognize and bind to pathogenic, primarily viral, nucleic acids. TLRs activate several complement pathways and signaling complexes, like MyD88 and TRIF, IRAK kinases, TRAF6, TAK1, and the IKK complex, which finally lead to the stimulation of transcription factors and the synthesis of proinflammatory cytokines (El-Zayat et al., 2019; Zhao et al., 2026). During sepsis, the TLR pathways are the major signaling initiators of the inflammatory response and serve as the first priming pathways of the inflammasome (Figure 2) (Wang et al., 2020).

#### 3.1.2 NOD-like receptors

NLRs are cytoplasmic PRRs that detect the invasion of microbes and the occurrence of stress in the cell (Arnold et al., 2018). NLRs such as NOD1, NOD2 and NLRP3 respond to a diverse array of stimuli, including ROS, mitochondrial dysfunction, extracellular ATP, crystalline substances, and various danger signals (Zhong et al., 2013). Among the various NLRs, the NLRP3 inflammasome is the most studied species due to its important role in the disease progression in sepsis (Almeida-da-Silva et al., 2023). Inflammasomes are formed when NLRP3 undergoes oligomerization with the adaptor protein ASC and procaspase-1 (Saavedra-Torres et al., 2025). Caspase-1 is activated to cleave the precursors of the interleukins, IL-1 and IL-18, to their mature active forms. In addition, gasdermin D is cleaved to induce pyroptosis. The inflammasome is also a result of the coordinated activation of the TLR-NF-κB signaling pathway, which is required for the transcriptional priming of NLRP3 and pro-IL-1β (Saavedra-Torres et al., 2025).

#### 3.1.3 Cytosolic DNA sensors

Detection of DNA that is misplaced in the cytosol is an essential aspect of recognizing and responding to some invasive pathogens and damaged or rogue cells (Zhou et al., 2023). The cGAS–STING pathway is the most well-researched system for identifying cytosolic DNA and is a pathway of some interest for the study of inflammation in sepsis (Hooftman et al., 2026). cGAS (cyclic GMP–AMP synthase) has a broad range for detecting cytosolic double-stranded DNA (dsDNA) regardless of whether that DNA is of microbial or host origin (Bushra et al., 2026). Binding of dsDNA leads to cGAS catalyzing the formation of cGAMP (cyclic GMP–AMP) (Decout et al., 2021). cGAMP is a signaling molecule that binds to and activates the STING protein that resides in the membrane of the ER (Han et al., 2024). Once activated, cGAS recruits and activates TBK1 (TANK-binding kinase 1), leading to the phosphorylation of IRF3 (Interferon regulatory factor 3) and the transcription and/or translation of type I interferons (Pan et al., 2023). STING also recruits and initiates the NF-κB pathway, which leads to the production of pro-inflammatory cytokines. During sepsis, mitochondrial degeneration releases mtDNA into the cytosol (Nakahira et al., 2011). cGAS recognizes mtDNA as an endogenous signal to activate the cGAS pathway (Liu Q. et al., 2021). Thus, cGAS–STING signaling contributes to antimicrobial defence while amplifying inflammation and organ injury (Khan et al., 2024).

#### 3.1.4 RIG-I-like receptors

RIG-I-like receptors (RLRs) are key cytoplasmic receptors capable of sensing distinct viral replication stages (Rehwinkel and Gack, 2020). RLRs include retinoic acid-inducible gene I (RIG-I), melanoma differentiation-associated protein 5 (MDA5), and laboratory of genetics and physiology 2 (LGP2). Viral RNA recognition triggers RIG-I and MDA5 binding to mitochondrial antiviral signaling protein (MAVS) and subsequent activation of TBK1, IRF3, IRF7, and NF-κB (Yoneyama et al., 2024). While the primary focus of RLR signaling is on antiviral defenses, many of the features and manifestations of viral sepsis and secondary viral infections are caused by the engagement of this pathway (van Huizen and Gack, 2025). Additionally, the signaling of MAVS has a wide network of interactions with multiple aspects of mitochondrial dynamics, oxidative stress, and inflammasome activation, contributing to a systemic inflammatory response (Lu Y. et al., 2025).

#### 3.1.5 C-type lectin receptors

C-Type lectin receptors (CLRs) bind to carbohydrate moieties of fungi, bacteria, parasites, and damaged host cells (Drouin et al., 2020). Examples include Dectin-1, Dectin-2, Mincle, and DC-SIGN (Malamud and Brown, 2024). Spleen tyrosine kinase (Syk) and the CARD9-BCL10-MALT1 signaling complex are recruited upon CLR activation, leading to NF-κB and MAPK pathway activation (Bermejo-Jambrina et al., 2018). CLR signaling is crucial for the defense against fungal sepsis and works in conjunction with TLR signaling to boost cytokine release, phagocytosis, and neutrophil recruitment (Hatinguais et al., 2023). CLR signaling has been shown to interact with inflammasome activation and influence the polarization of the adaptive immune response (Zhang Y. et al., 2026; Geijtenbeek and Gringhuis, 2009).

### 3.2 Signal integration in innate immunity

An essential feature of innate immunity is the synthesis of signals that emanate from multiple PRRs rather than the activation of distinct pathways (Qian C. et al., 2026). Synthesis of signals occurs as a result of interaction between TLRs, NLRs, the cGAS–STING and RLR pathways, and CLRs (Wiersinga and van der Poll, 2026). Shared adaptor proteins, kinases, and transcriptional regulators are implicated in mediating feedback across signaling pathways. MyD88, TRIF, and TRAF6 are prominent signaling hubs (Lee et al., 2023; Qian C. et al., 2026). Among the multitude of innate immune signaling pathways, NF-κB is the most prominent pathway that integrates the synthesis of signals in inflammatory cytokine production, the recruitment of leukocytes, the activation of endothelial cells, and cellular survival. IRFs are involved in the synchronization of pathways that elicit antiviral responses (Qian C. et al., 2026; Wang L. X. et al., 2025; Wang L. et al., 2025). The maturation of IL-1β and IL-18, accompanied by pyroptosis, is also a result of the activation of inflammasomes (Heinke, 2025). Calcium, ROS from mitochondria, and additional factors provide feedback across pathways to create a decentralized network that regulates immune responses based on the level of stress and injury to tissues and the pathogenic load (Patergnani et al., 2021). In sepsis, constant stimulation by microbial PAMPs, along with host-derived DAMPs, disrupts homeostasis of immune regulation (Hossan et al., 2026). Ultimately, over-activation of immune pathways amplifies inflammatory signaling. Conversely, sustained immune stimulation leads to immune exhaustion and suppression (Ovali and Percin, 2025; Hemati and Mohsenipour, 2026). Therefore, the first approach to untangle the integrated structure of signaling pathways of innate immunity will be to understand the patterns of the interconnection of TLR, NLRP3 inflammasome, cGAS–STING, and NF-κB pathways (Pan et al., 2023; Pan et al., 2024). This will be the main focus of the following sections of this review.

## 4 Molecular crosstalk among the signaling pathways

### 4.1 TLR–NF-κb signaling axis

TLRs are the first receptors of infection at the molecular level and are the primary activators of inflammatory signaling (Qian C. et al., 2026). TLRs recognize PAMPs and DAMPs and recruit the adaptor proteins MyD88 and TRIF to activate kinases and the NF-κB inhibitory kinase (IKK) complex. This leads to the translocation of NF-κB and the synthesis of inflammatory proteins (Hooftman et al., 2026). NF-κB causes the synthesis of pro-inflammatory mediators such as TNF-α, IL-1β, IL-6, CXCL8, CCL2, adhesion molecules, COX-2, iNOS, NLRP3, and Pro-IL-1β (Almeida-da-Silva et al., 2023). This is how TLR signaling prompts the synthesis of the components of the immune system (Kox et al., 2026). TLR signaling is not only responsible for the formation of pro-inflammatory mediators but also for the production of feedback regulators (Figure 2) (Chen Y. et al., 2025; Wang H. et al., 2026). This is important, as it allows the amplification and control of the end of the inflammation process (Liu et al., 2025).

### 4.2 TLR–NLRP3 inflammasome interactions

**FIGURE 3:**
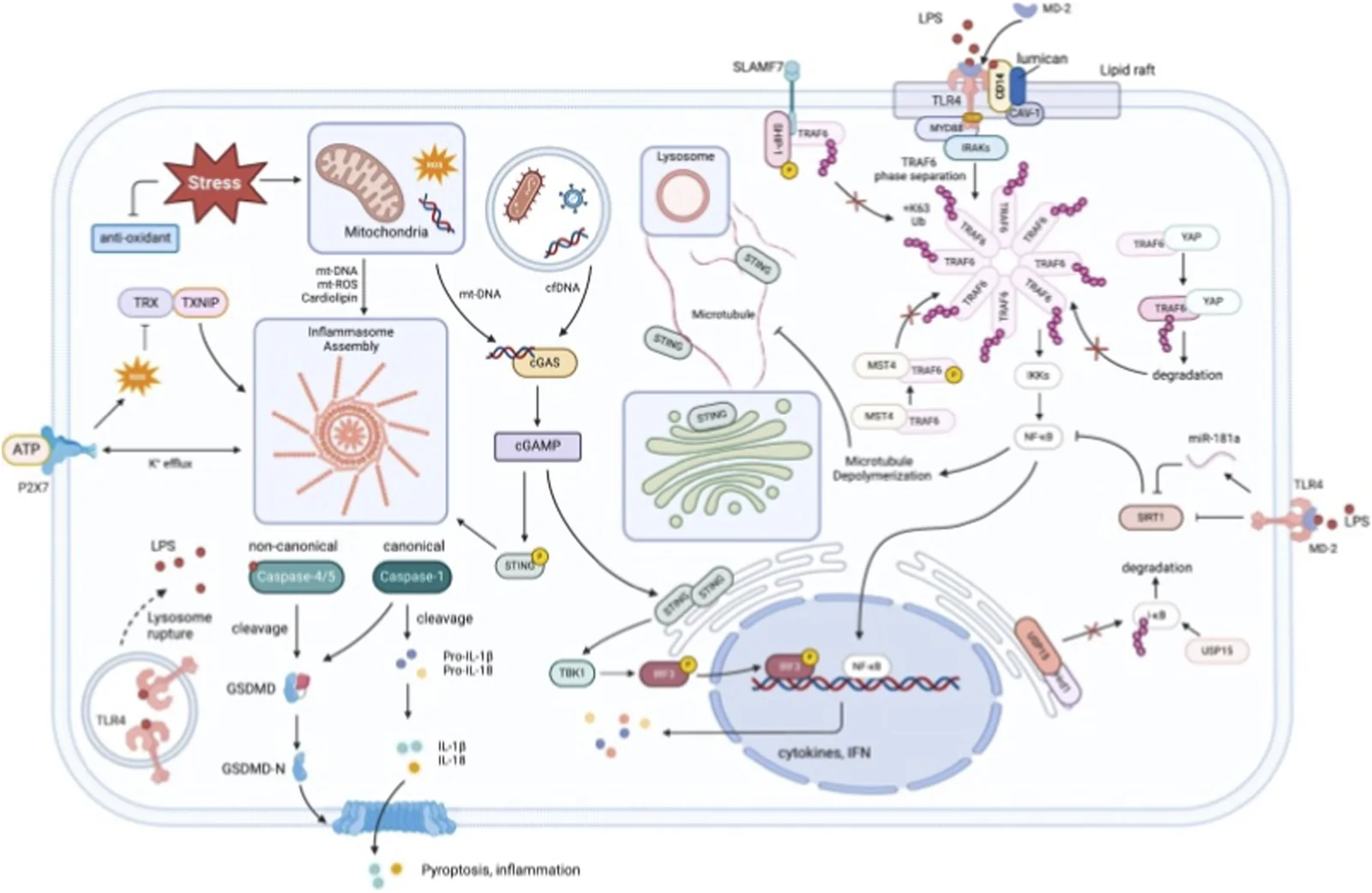
NLRP3 inflammasome and NF-κB crosstalk in sepsis. In the pathway, LPS binding stimulates TLR4 dimerization and activates downstream mediators, MYD88 and IRAKs, resulting in K63-linked TRAF6 ubiquitination and phase separation. The NF-κB pathway is activated, and IL-18 and other cytokine levels are increased. SLAMF7, SHIP-1, MST4, and YAP may act on TRAF6 to restrict K63 ubiquitination. On the other hand, mtDNA from damaged mitochondria binds to cGAS, promoting the formation of the NLRP3 and ending up in the generation and release of IL-1β and IL-18. SLAMF7, SHIP-1, MST4, and YAP may act on TRAF6 to restrict K63 ubiquitination. On the other hand, mtDNA from damaged mitochondria binds to cGAS, promoting the formation of the NLRP3 and ending up in the generation and release of IL-1β and IL-18. Reprinted from Experimental and Molecular Pathology, Volume 142 June 2025, 104,963, Yuehua L et al., “ Advances in sepsis research: Insights into signaling pathways, organ failure, and emerging intervention strategies”, Copyright (2025), with permission from Elsevier.

The interaction between TLR signaling and the NLRP3 inflammasome is the most well-studied interaction of all the innate immune system components (Qian C. et al., 2026; Huang et al., 2021). In the priming phase, the activation of NF-κB by TLRs provides the first signal for the priming of the NLRP3 and the transcription of Pro-IL-1β and Pro-IL-18 (Kumar, 2020a; Shi et al., 2021). This signifies the first step of the transcriptional priming, as the inflammasome would not respond to the signals of activation (Wang W. et al., 2025). Priming occurs by a variety of cellular and mitochondrial signals such as ROS, release of ATP, efflux of potassium, calcium influx, as well as rupture of lysosomes and oxidized mtDNA (Bushra et al., 2026; Zhang et al., 2024). The assembly is the first step in the process of cytotoxin-induced cell death via the cleavage of Pro-IL-1β, Pro-IL-18, and gasdermin D by activated caspase-1 (Chen et al., 2018). IL-1β released from the cell binds to receptors on neighboring immune cells and further activates NF-κB signaling. DAMPs released from pyroptotic cells further activate TLRs and propagate the feedback loop (Figure 3) (Danielski et al., 2020; Dubyak et al., 2023).

### 4.3 cGAS–STING–NF-κb crosstalk

**FIGURE 4:**
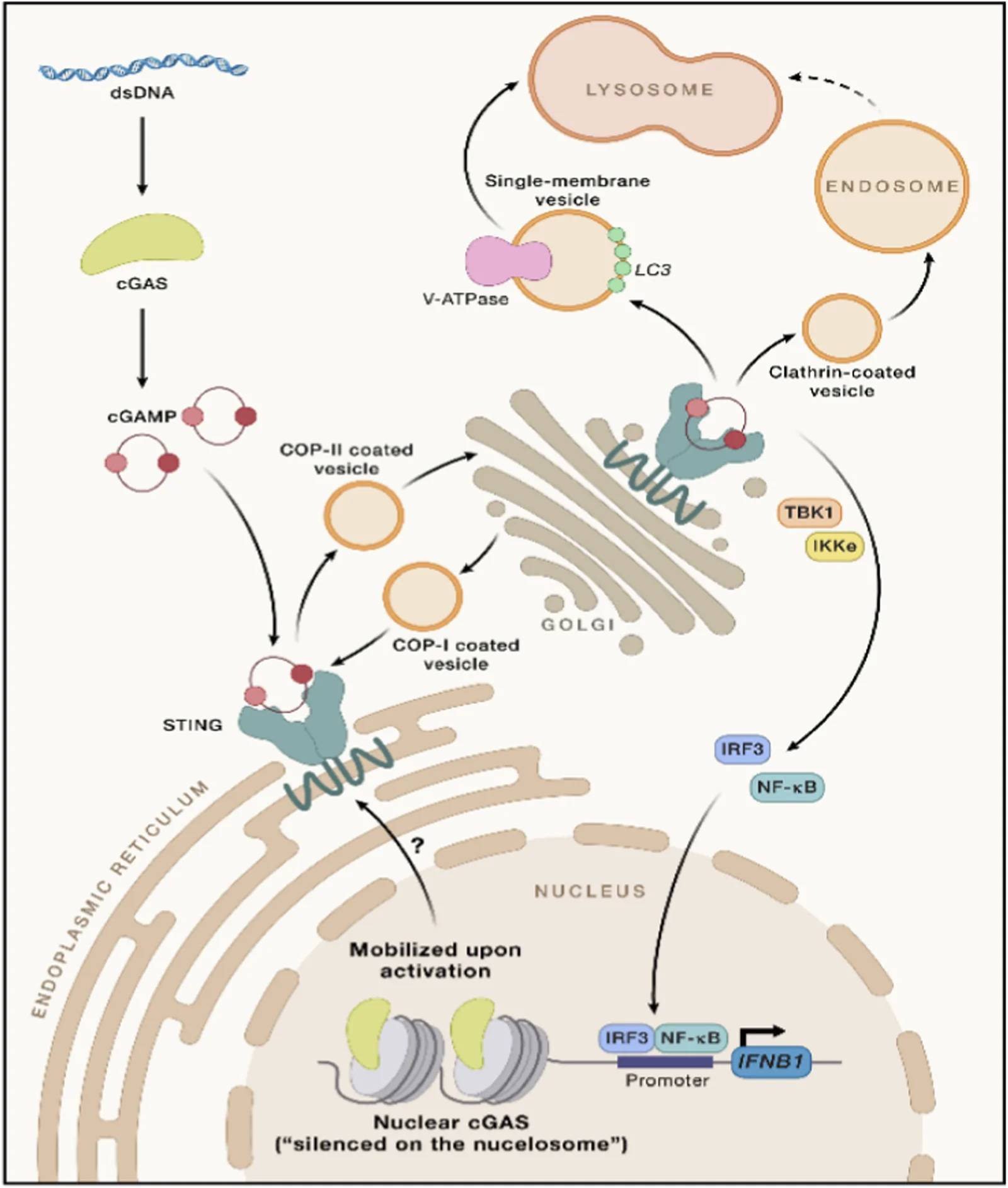
The cGAS-STING signaling cascade. The binding of dsDNA with cGAS results in the formation of c-GAMP, activating the STING in the ER. STING, on oligomerization, translocates into the Golgi, wherein activated STING recruits TBK1, and signaling leading towards the phosphorylation and translocation of IRF3 into the nucleus. STING also activates NF-κB through TBK1 and IKKε, and this concerted effort results in the expression of type I interferons, pro-inflammatory cytokines, chemokines, and other antiviral modulators. STING is later trafficked towards the endolysosomal system for degradation. Reprinted from Cell, Volume 189, Issue 13, 25 June 2026, Pages 3849–3870, Alexander H et al., “The cGAS-STING pathway: Mechanism and medical implications”, Copyright (2026), with permission from Cell press.

The cGAS–STING pathway links the sensing of cytosolic DNA to other inflammatory signaling pathways (Zhou et al., 2026). The detection of cytoplasmic microbes or mtDNA activates cGAS, and this recognizes the presence of cytoplasmic DNA (Zhou et al., 2023). cGAS then synthesizes cGAMP and activates STING, which further activates TBK1 and the IKK complex (Wang W. et al., 2025). This is a dual signaling pathway that activates IRF3 and NF-κB, thus producing type I interferons and pro-inflammatory cytokines and chemokines. The cGAS–STING pathway is used for the induction of adaptive immune and inflammatory responses (Pan et al., 2023). NF-κB-mediated inflammation causes injury to mitochondria and combines with oxidative stress and necrosis to increase the release of cytoplasmic DNA (Liu et al., 2025). This further activates cGAS. This type of regulatory relationship is the most important mechanism for the persistent inflammatory response associated with severe sepsis (Figure 4) (Decout et al., 2021).

### 4.4 cGAS–STING–NLRP3 inflammasome crosstalk

Recent advances have shown that there is a high degree of interconnectivity and communication between the cGAS–STING-NLRP3 axes (Saavedra-Torres et al., 2025). During sepsis, damaged mitochondria release mtDNA, and this simultaneously activates cGAS, TLR9, and the NLRP3 inflammasome (Zhong et al., 2016). Therefore, damaged mitochondria are one of the primary molecular events that connect many innate immune pathways (Liao et al., 2026). Active STING promotes the activation of the inflammasome through several NF-κB-mediated priming, induction of mitochondrial ROS, lysosomal dysfunction, and ER stress (Man et al., 2017). The pyroptotic cell death caused by the NLRP3 inflammasome releases mitochondrial and nuclear DNA, ATP, HMGB1, and other DAMPs and further activates the cGAS–STING pathway (Zheng et al., 2024). This creates a self-sustaining inflammatory circuit that causes injury to multiple organs in sepsis (Zhang et al., 2020a).

### 4.5 NF-κb as master integrator of innate immune signaling

NF-κB is the central mediator of innate immune signaling networks, as it integrates signals from almost all recognized PRRs (Hoffmann et al., 2025). NF-κB is activated by TLRs, NOD receptors, cGAS, STING, cytokine and complement receptors, and pathways of oxidative stress (Dorrington and Fraser, 2019). Once activated, NF-κB is responsible for the transcription of genes for cytokines, chemokines, components of the inflammasome, adhesion molecules, coagulation factors, antimicrobial proteins, regulators of cell survival, and enzymes of metabolism (Iacobazzi et al., 2023). As NF-κB is responsible for the initiation of inflammation and the priming of the inflammasome, it is the molecular link between the recognition of innate immunity and the response of inflammation x (Fu et al., 2026).

### 4.6 Integrated molecular network driving sepsis progression

**FIGURE 5:**
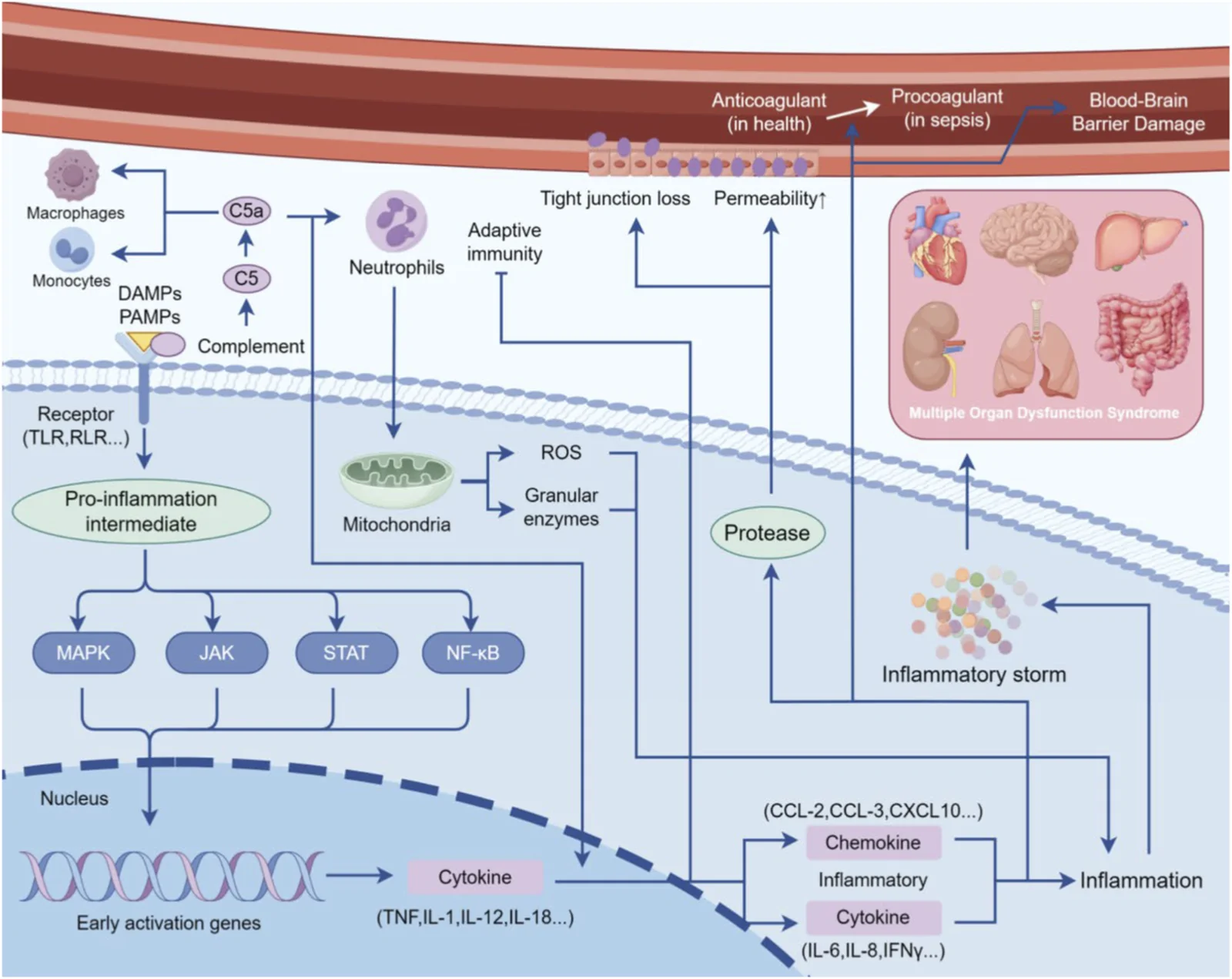
The integrated inflammatory network driving sepsis progression. The figure illustrates the major components of the integrated inflammatory network involving TLR–NLRP3–cGAS-STING–NF-κB, their signaling pathways, and their roles in the progression of sepsis (Wu H. et al., 2025). Reprinted from Cellular & Molecular Biology Letters, Volume 30, 15 August 2025, Huang W et al., “ Cell death signaling and immune regulation: new perspectives on targeted therapy for sepsis”, Copyright (2025), with permission from Springer Nature.

TLR, NLRP3, cGAS-STING, and NF-κB signaling pathways do not act alone, and instead, appear to act as a coordinated molecular network with multiple reciprocal regulatory loops (Gai et al., 2026). TLRs recognize the first signals of an invading pathogen and also activate NF-κB to begin the inflammatory response and the priming of the inflammasome (Yilmaz et al., 2026). NLRP3 and the cGAS-STING axis, which are both activated during infection, cause cellular stress that leads to the release of ATP and mtDNA and the onset of oxidative stress (Addissouky et al., 2023). Pyroptosis and cell damage also lead to the release of IL-1β, IL-18, type I interferons, and DAMPs, which sustain TLR and NF-κB signaling and the self-reinforcing cycle of inflammation (Iacobazzi et al., 2023). Although efficient pathogen clearance is promoted by the coordinated network even at the early stages of infection, pathological overactivation of the network leads to excessive inflammation, damage to the endothelium, and organ failure (Zheng et al., 2024). On the contrary, chronic activation of the network leads to immune suppression and an enhanced risk of secondary infections due to immune exhaustion, metabolic dysregulation, negative feedback, and epigenetic changes (Kumar, 2020a). The available evidence suggests that the multiple overlapping networks of TLRs, NLRP3, cGAS-STING, and NF-κB are the dominant features of sepsis as opposed to a few disparate network signaling pathways (Foley et al., 2015; Sun et al., 2025). This perspective is a rationale for the next-generation of sepsis treatment aimed at protecting immune balance and controlling sepsis by selectively modulating signaling crosstalk, as opposed to treating with modulators of inflammatory mediators (Figure 5) (Yilmaz et al., 2026; Wu H. et al., 2025).

## 5 Modulators of signaling crosstalk

### 5.1 Mitochondrial dysfunction and ROS

**FIGURE 6:**
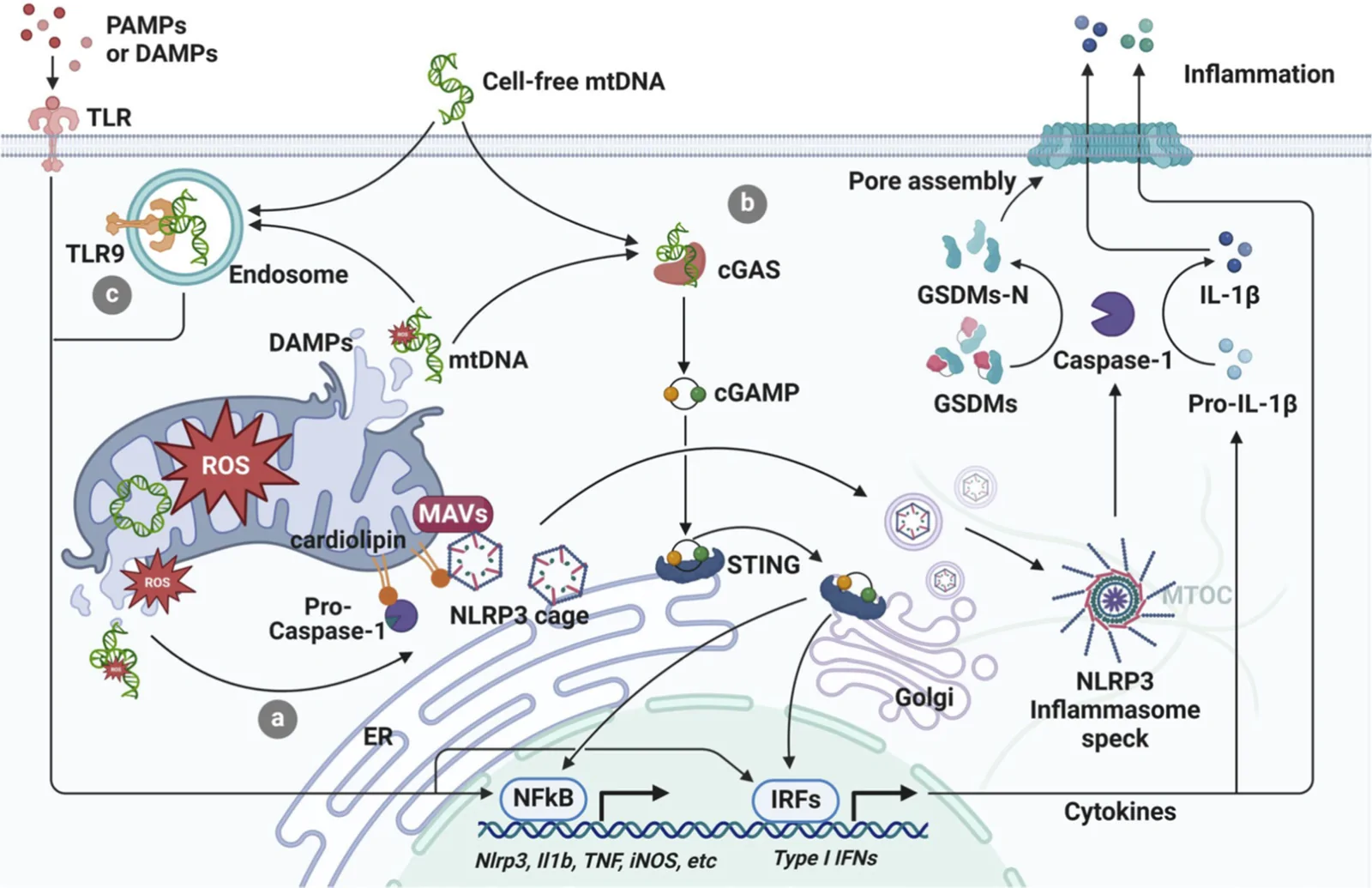
Inflammatory pathways in mitochondria-regulated sepsis. PAMPs from microbes or DAMPs from damaged host cells recognize TLRs. In **(a)** NLRP3 inflammasome activation occurs by mtROS and ox-mtDNA released from damaged mitochondria. MAVS and cardiolipin facilitate the spatial association between mitochondria and NLRP3 for recognizing the inflammatory signals from the damaged mitochondria by NLRP3. Activated NLRP3 facilitates the formation of caspase-1, which processes GSDMs and pro-inflammatory cytokines. The mature cytokines are released from the GSDMs’ pores to spread inflammatory signals. In **(b)** mtDNA binds cGAS, and the activated complex facilitates the synthesis of cGAMP, which in turn causes a change in STING, which is activated at the ER. Activated STING after translocation to the Golgi triggers the expression of IRF3 and NF-κB to start the synthesis of type I interferons and other pro-inflammatory cytokines required for the host-pathogen response. In **(c)**, mtDNA, due to its similarity with bacterial DNA, is recognized by TLR9. As with other TLRs and the cGAS- STING axis, the TLR9 pathway activates IRF3 and NF-κB to modulate the inflammatory response (Hu D. et al., 2024). Reprinted from Critical Care, Volume 28, 03 September 2024, Dongxue H et al., “ Mitochondrial dysfunction in sepsis: mechanisms and therapeutic perspectives”, Copyright (2024), with permission from Springer Nature.

Mitochondria are more than cellular powerhouses; they are important mediators of innate immune system signaling (Ji et al., 2026). In sepsis, a combination of proinflammatory stimuli, oxidative stress, calcium overload, and impaired mitochondrial quality control results in a specific form of systemic mitochondrial dysfunction that produces a state of energy crisis (depletion of ATP), increased mitochondrial permeability, disorganization of inner membrane integrity, and excessive mitochondrial production of ROS (Figure 6 **)** (Nedel et al., 2023; Chen X. et al., 2026). Mitochondrial-derived ROS act as secondary signaling molecules and promote the dysregulation and amplification of multiple inflammatory pathways (Chen X. S. et al., 2026).

Among the many effects of elevated mitochondrial ROS is the activation of the NLRP3, which is mediated by the promotion of structural change and assembly of the inflammasome complex (Ustundag, 2026). In addition, stress-induced ROS and oxidative damage to mitochondria facilitate the release of mtDNA, which in turn acts as a proinflammatory signal by activating the cGAS-STING cascade and the TLR9 defense pathway (Cao Y. et al., 2025). mtDNA released into circulation activates several interconnected innate immune responses (Yang T. L. et al., 2026). Uncontrolled inflammation initiates and maintains self-perpetuating inflammatory pathways that further tissue injury and increase apoptosis, endothelial dysfunction, and pyroptosis (Li et al., 2026). Nonetheless, the harmful effects of excessive ROS and the disparity between proinflammatory and anti-inflammatory defenses can diminish the inflammatory damage associated with sepsis (Liu and Liang, 2025). While the potentially beneficial effects comprise the activation of the Nrf2 signaling pathway. The generation of mitochondrial oxidative stress has become a valid target for therapeutic interventions in the treatment of sepsis (Figure 6) (Wang W. et al., 2025).

### 5.2 Autophagy and mitophagy

Autophagy is an intracellular process that removes damaged organelles, protein aggregates, and intercellular pathogens to help maintain cellular homeostasis (Vargas et al., 2023). With respect to sepsis, autophagy negatively regulates innate immune signaling, mitigating hyper-inflammation (Iba et al., 2024). Mitophagy specifically removes damaged mitochondria via selective autophagy. It is important to remove mitochondria that are producing high levels of ROS and releasing mtDNA from the cell (Nedel et al., 2023). Mitophagy is effective in neutralizing the NLRP3 - cGAS–STING axis and in mitigating the effects of NF-κB. Autophagy also removes the NLRP3 and ASC inflammasome components, clears cytoplasmic DNA, and STING, along with DAMPs (Liu Q. et al., 2021). During severe sepsis, defective autophagy results in continuous inflammation, increased pyroptosis, and organ injury due to mitochondrial dysfunction (Roca-Agujetas et al., 2019). Important autophagy regulators include AMP-activated protein kinase, mTOR, Beclin-1, ULK1, and TFEB. These regulators also impact innate immune signaling and cellular quality-control systems in a coordinated manner (Kimura et al., 2017; Mercer and Tooze, 2021; Zhao et al., 2020).

### 5.3 Immunometabolic reprogramming

The activated innate immune cells induce the reprogramming of metabolism to adapt to the new functions (Fitzpatrick, 2019; Xian et al., 2025). Metabolic reprogramming of macrophages, neutrophils, dendritic cells, and endothelial cells during sepsis is characterized by the dominant use of aerobic glycolysis, increased glutaminolysis, changes in fatty acid metabolism, and impairment of cell respiration and sepsis-related inflammation (Liu W. et al., 2023; Wu et al., 2026). Activated glycolysis not only provides ATP but also generates intermediates of metabolism that are necessary for biosynthesis in response to infections and inflammation (Iacobazzi et al., 2023). Products and enzymes of the glycolytic pathway can influence and control the activation of NF-κB and NLRP3, as well as the production of cytokines. Some of the metabolites of the TCA cycle can act as signaling molecules (Willmann and Moita, 2024). For example, succinate can stabilize HIF-1α and can lead to the synthesis of IL-1β and the activation of NLRP3; citrate can facilitate the production of fatty acids, prostaglandins, and nitric oxide; fumarate can induce active changes to the structure of chromatin in relation to transcriptional control of inflammatory genes, while itaconate can act as a strong anti-inflammatory metabolite and inhibit succinate dehydrogenase, NF-κB and NLRP3 signaling (Kelly and O'Neill, 2015; Liu et al., 2024; Yuan et al., 2026). Likewise, fatty acid oxidation can promote alternative anti-inflammatory macrophages. Disruption of fatty acid metabolism can promote inflammation and increase oxidative stress (Fitzpatrick, 2019). Cholesterol crystals and oxidized phospholipids can activate NLRP3 against the dysregulation of metabolism and immune stimulation. Metabolic inflammation is best described as an insufficient energetic coupling between metabolism and inflammation.

### 5.4 Epigenetic regulation

The contributions of epigenetic changes can manifest as long-term inflammation (Falcao-Holanda et al., 2021). Systemic inflammation can lead to a severe lack of immune regulation as a result of epigenetic changes (Li Y. et al., 2021). Some examples of major epigenetic changes are: (i) Changes in the activity of DNA methyltransferases can result in the methylation of specific promoter regions of inflammatory genes, leading to modified synthesis of cytokines and the tolerance of the immune system (Lopez-Cruz et al., 2024; Pignataro et al., 2025). (ii) Changes in histone acetylation, methylation, and phosphorylation can lead to changes in the accessibility of chromatin and the transcription of inflammatory genes (Weiterer et al., 2015; Xu Y. F. et al., 2025). Histone acetylation is primarily controlled by NF-κB and is a major factor in the regulation of inflammatory gene expression (Wu D. et al., 2023). (iii) Chromatin remodeling complexes that use ATP are known to alter the accessibility of NF-κB, IRF3, and STATs and other factors to promoters of genes linked with inflammation (Pignataro et al., 2025; Tartey and Takeuchi, 2015). Epigenetic modifications and chromatin remodeling represent key mechanisms underlying endotoxin tolerance, trained immunity, immunological exhaustion and long-term immune dysregulation in sepsis survivors (Marcu A, 2024) (Wang H. et al., 2026; Zhang S. et al., 2020).

### 5.5 Non-coding RNAs

The role of non-coding RNAs in controlling innate immunity at the level of signaling and beyond, including at the level of PTMs, is an area of intense research (Zhang et al., 2017). miRNAs modulate inflammatory responses through the post-transcriptional silencing of mRNA (Xie et al., 2026). The main miRNAs studied in sepsis are: miR-146a, which targets IRAK1 and TRAF6 to inhibit TLR-NF-κB pathway activation (Valsamaki et al., 2024); miR-155, which targets negative regulators of NF-κB to promote inflammatory cytokines (Ghafouri-Fard et al., 2021); miR-223, which inhibits NLRP3 and inflammasome activation; miR-21 (Formosa et al., 2022), which enhances the production of pro-inflammatory cytokines and regulates macrophage polarization, and miR-125b, which modulates immune cell activation and TNF-α production (Bindayna, 2024). Long non-coding RNAs (lncRNAs) affect transcription, chromatin, and RNA stability and act at the protein level (Wu H. et al., 2020; Xue et al., 2026). A number of lncRNAs are known to affect NF-κB activation, inflammasome assembly, and the type I interferon pathway (Wang et al., 2021). Circular RNAs (circRNAs) are known to regulate TLR, NLRP3, cGAS-STING, and NF-κB signalling pathways by acting as miRNA sponges (Beltran-Garcia et al., 2020; Liu Q. et al., 2026). Current studies indicate that circRNAs may participate in immune system adaptation and may be useful as sepsis-related severity biomarkers (Zhong B. et al., 2024).

### 5.6 Cytokines, chemokines and endocrine regulation

Inflammatory cytokines represent final pathway products of innate immune system activation but may also represent important first pathway products of cross-talk (Tuerxun et al., 2026; Gharamti et al., 2022). TNF-α, IL-1β, and IL-6 stimulate NF-κB signaling via autocrine and paracrine activation (Doganyigit et al., 2022). In addition, IL-1β signaling promotes the priming of inflammasomes. Interferons of type I, as a product of cGAS-STING macrophage activation and adaptive immunity (Zhang Y. et al., 2026). In contrast, anti-inflammatory cytokines such as IL-10 and TGF-β suppress NF-κB activation, reduce inflammasome and antigen-presenting activities, and support the resolution of inflammation (You et al., 2025). The progression of sepsis is predisposed by the interplay of pro-inflammatory and anti-inflammatory cytokines. During sepsis, the systemic inflammatory response is controlled by neuroendocrine mechanisms (Fan et al., 2025; Widmer and Schuetz, 2018). Glucocorticoids control NF-κB by promoting IκBα and decreasing the transcription of pro-inflammatory cytokine genes while enhancing the transcription of anti-inflammatory genes (Mehdi et al., 2025). Catecholamines and the anti-inflammatory cholinergic pathway, which functions through α7 nicotinic acetylcholine receptors, modulate macrophage metabolism and differentiation (Reddy et al., 2024). Insulin, leptin, ghrelin, vitamin D, and other hormones, in addition to the glucocorticoids, regulate the immune response by interacting with metabolism and other pathways (Vella et al., 2025; Melis et al., 2023). A summary of cytokines and their properties is mentioned below (Table 2).

**TABLE 2:** Selected properties of some important cytokines in the pathogenesis of sepsis (Vella et al., 2025).

Class/Type	Selected members	Key functions and remarks
Interleukins	IL-1α, IL-1β, IL-18, IL-33, IL-36 (proinflammatory) IL-1RA, IL-4, IL-10, IL-37 (anti-inflammatory); IL-6 (dual); IL-3	Innate immunity activation, inflammation regulation. IL-6: Dual behavior depending on receptor (proinflammatory via IL-6R, anti-inflammatory via IL-6Rα). IL-3 is involved in cytokine storm regulation during sepsis
Tumor Necrosis Factors (TNF)	TNF-α, LT-α, LT-β, FasL, CD40L, CD30L, CD27L, TRAIL/apo-2L	Modulation of cellular responses, inflammation Apoptosis. TNF-α: Produced by monocytes/macrophages; activates NF-kB, JNK, ERK pathways; involved in autoimmune, cardiovascular, and inflammatory diseases
Interferons (IFNs)	IFN-γ (type II)	Antiviral defense, activation of innate and adaptive immunity. IFN-g: Activates macrophages; induces IL-6, TNF-α, IL-10 production via JAK-STAT; IFNγR1 deficiency increases infection susceptibility
Complement System	CCL1, CCL2, CCL8, CCL20 (CC); CXCL8, CXCL10, CXCL12 (CXC)	Leukocyte migration, inflammation signaling. Key roles in sepsis severity and prognosis; CXCR2 regulates neutrophil recruitment; upregulated in cytokine storm and ARDS
Colony-stimulating Factors (CSFs)	GM-CSF, G-csf, m-csf	Myeloid cell differentiation and activation. GM-CSF: Promotes both pro-inflammatory and anti-inflammatory cascades; modulates survival and function of various immune cells; impact depends on individual immune status
Transforming Growth factors (TGFs)	TGF-β1, β2, β3	Immunosuppression, cell proliferation, and differentiation. Promotes tregs with IL-2, or Th17 cells with IL-6/IL-21; excess TGFβ impairs early immune responses and suppresses T/B cells (except Tregs)

### 5.7 Gut microbiota and microbial metabolites

The gut microbiota serve as a vital mediator of systemic immune responses in sepsis (Wiersinga and van der Poll, 2026). Translocation of gut microbiota or damage to the gut barrier may result in the appearance of LPS, peptidoglycan, and other products of microbial metabolism in the bloodstream (Li et al., 2022; Hu J. et al., 2024). These and other microbial metabolites stimulate innate immunity and inflammation (Yumoto et al., 2026). Short-chain fatty acids (SCFAs) and other metabolites have anti-inflammatory effects and improve the gut barrier (Adame et al., 2026; Wu J. et al., 2023). Dysbiosis of the gut contributes to inflammation and immune responses that leave a sepsis patient vulnerable to subsequent infections (Piccioni et al., 2024).

### 5.8 Genetic and post-translational regulation

Natural variations in human genes differentially impact sepsis and the immune response (Engoren et al., 2022). Single nucleotide polymorphism (SNP) studies have shown differential clinical presentations and outcomes for polymorphisms of genes that encode TLRs, NLRP3, STING, components of NF-κB, cytokines, and associated adaptor proteins (Qian C. et al., 2026; Holmes et al., 2003). Despite these genetic variations, innate immune response signaling is differentially regulated by PTMs of signaling proteins within immune response networks (Qian C. et al., 2026; Wang Y. et al., 2026). These modifications include phosphorylation (Hooftman et al., 2026), ubiquitination (Liu T. et al., 2023; Kim et al., 2024), SUMOylation (Cai J. et al., 2026), lactylation (Chen Y. et al., 2025; Fang et al., 2026), acetylation (Xu Y. F. et al., 2025), palmitoylation (Cao S. et al., 2025), glycosylation (Staudacher et al., 2022), and proteolytic cleavage. These modifications impact the dynamics of networks and the response to and the resolution of inflammation (Yang et al., 2025). Dysregulation of these PTMs is central to chronic activation of the TLR, NLRP3, cGAS–STING, and NF-κB signaling cascades during sepsis (Song et al., 2024).

### 5.9 Therapeutic strategies targeting signaling pathway modulators

The understanding that innates immune responses and their regulation involve a multitude of interconnecting signaling pathways has shifted the focus of sepsis therapies away from addressing single pathways to ‘network’ therapies (Zhang and Ning, 2021; Cui et al., 2024). Therapeutic strategies that focus on the modification of intramitochondrial signaling, alleviating mitochondrial oxidative stress, immunometabolic signaling and the regulation of inflammation and autophagy, such as epigenetic regulation and modification of pathogenic ncRNAs, have shown potential in preclinical studies of sepsis (Wu H. et al., 2025; Xie et al., 2026; Ivashkiv, 2018; Tian et al., 2023). Of significance to these pathways is that most of them converge at a common signalling point. For example, AMPK activation potentiates autophagy and improves mitochondrial function while concurrently suppressing NF-kB, inflammasome complex signaling, and cGAS–STING signaling (Li Y. et al., 2025). Likewise, the stimulation of mitophagy exemplifies the inhibition of several inflammatory signaling pathways at a common, upstream, inflammatory signaling source (Yilmaz et al., 2026; Kim et al., 2016). Further, sepsis treatment approaches will likely integrate several new biotherapeutics, alongside established, integrated immunometabolic, epigenetic, and microbiome therapies, as well as conventional antimicrobials (Qian C. et al., 2026; Marques et al., 2023). These multifunctional, biotherapeutic approaches focus on the crosstalk, intercommunication, and functional balance of innate immune system signaling pathways, as opposed to the more traditional focus on the individual components of the inflammatory process (Tuerxun et al., 2026). It may offer the possibility of restoring the balance of immune system function, preventing multiple organ failure, and increasing survival for sepsis patients (Zhang et al., 2020c; Jiang W. et al., 2026).

## 6 Crosstalk among other molecular pathways

### 6.1 MAPK signaling

Mitogen-activated protein kinase (MAPK) pathways consist of three major signaling pathways: ERK1/2, JNK, and p38 MAPK. JNK and p38 MAPK are activated by cytokines, TLRs, and receptors for growth factors through TAK1 and MAP kinase kinases (MKKs) (Pua et al., 2022). MAPKs, once activated, regulate the AP-1, CREB, ATF2, and Elk-1 transcription factors. MAPK signaling, along with NF-κB, modulates the transcription of inflammatory genes (Pei et al., 2025). ERK signaling promotes cell proliferation and cell survival primarily (Roux and Blenis, 2004). JNK and p38 MAPK signpost mainly cytokine production, cell death, oxidative stress, and the polarization of macrophages (Peng et al., 2014). p38 MAPK signaling plays a major role in the production of the inflammatory cytokines TNF-α, IL-1β, and IL-6, and JNK MAPK signaling induces mitochondrial damage and programmed cell death (Huang et al., 2009). MAPK signaling and the NLRP3 create positive feedback loops of inflammation by inducing the priming of the inflammasome and cytokine production (Diao et al., 2026). MAPKs, through the regulation of the interferons and inflammatory transcription, also shape cGAS–STING signaling (Jones et al., 2025).

### 6.2 PI3K/akt/mTOR signaling

This pathway is responsible for the regulation of multiple processes, including cellular metabolism, survival, and the proliferation of immune cells through the regulation of autophagy (Fattahi et al., 2022). PI3K activates the phosphorylation of inositol and brings about the formation of the lipid PIP3, which promotes the activation of Akt. Akt then phosphorylates several key cellular components, including mTORC1, GSK3β, and the FOXO transcription factors. Within innate immunity, PI3K/Akt signaling acts as a homeostatic regulator by limiting TLR-driven NF-κB activation while promoting the survival of innate immune cells (Sheng et al., 2026). The sepsis-associated persistent activation of this pathway may contribute to immune suppression by preventing antimicrobial immunity. mTOR, as a metabolic pathway integrator of cell growth, mediated by glycolysis, protein synthesis, and mitochondrial activity, is also responsible for the regulation of autophagy (Cui et al., 2024; Biasizzo and Kopitar-Jerala, 2020). Excessive mTOR activity results in the blockade of autophagy and the buildup of damaged mitochondria, higher levels of ROS, enhanced release of mtDNA and exaggerated activation of the NLRP3 and cGAS-STING pathways (Zhong H. et al., 2024). Thus, the simultaneous activation of the PI3K/Akt/mTOR pathway during sepsis is of critical importance to the integration of immune responses and cellular metabolism (Omolekan et al., 2024).

### 6.3 AMPK signaling

AMP-activated protein kinase (AMPK) is the most studied energy sensor. It is activated by an elevated AMP/ATP ratio during a shift to a more catabolic state (Jin et al., 2020). The activation of AMPK promotes various responses, including fatty acid oxidation, autophagy, mitophagy, and the induction of antioxidant defense and biogenesis of mitochondria (Liu et al., 2016). AMPK activity is linked with the inhibition of mTOR-driven anabolic pathways and innate immune signaling (Huang et al., 2018). AMPK is also vital in the mediation of mitogenic and pro-inflammatory forms of signaling and may be a promising therapeutic target (Yumoto and Coopersmith, 2024).

### 6.4 JAK–STAT pathway

**FIGURE 7:**
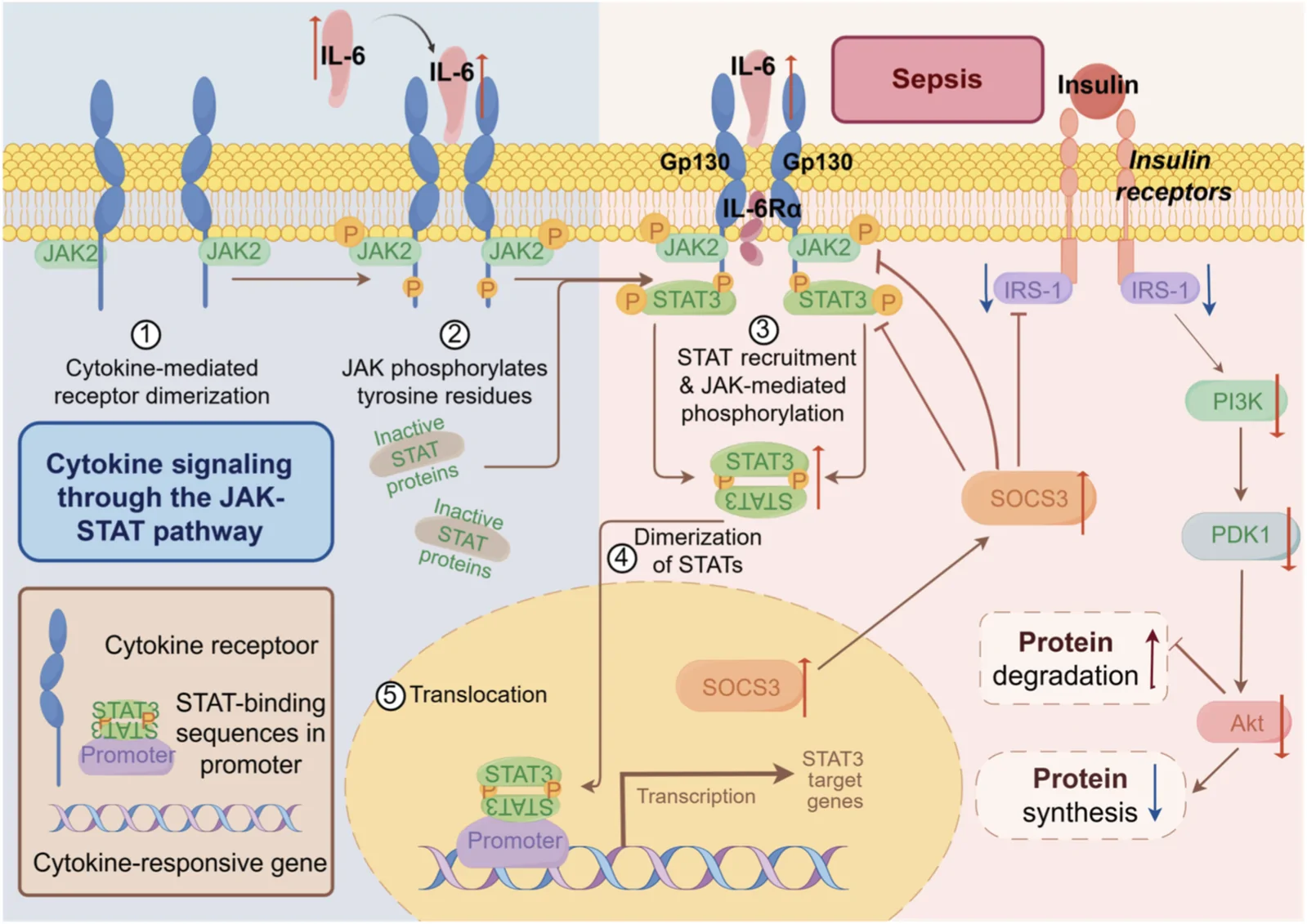
JAK/STAT signaling pathway in the pathophysiology of sepsis. Describe the main components of the JAK/STAT pathway in the figure (such as cytokine receptors, JAK1/2/TYK2, STAT1/3/6, ISGs, etc.) and their interaction with NF-κB in sepsis. Reprinted from Research, 1; 8:0,811, Tang L et al., “Decoding Sepsis: Unraveling Key Signaling Pathways for Targeted Therapies”, Copyright (2025), with permission from SPJ.

The JAK-STAT pathway is one of the most important and best understood signal transduction pathways utilized by many immunoregulatory cytokines and growth factors (Chen X. S. et al., 2026). The cytokines-receptors complex formation, such as IL-6, IFN-γ, IFN-α/β, IL-10, and GM-CSF, leads to the activation of JAKs and subsequent phosphorylation of STATs (Djidjik et al., 2025; Mahjoor et al., 2023). Phosphorylated STATs translocate to the nucleus and trigger transcription of targeted genes (Hu et al., 2023). During sepsis, the roles of the various STATs include (Ezeonwumelu et al., 2021): mediation of the cGAS-STING cascade and interferon response by STAT1; regulation of IL-6 signalling, immunosuppression, and the acute-phase response by STAT3; and promotion of macrophage polarisation and STAT6-mediated anti-inflammation (Cai et al., 2015). There is extensive interplay between JAK–STAT and NF-κB signaling. When IL-10 is present, STAT3 is activated, and NF-κB is suppressed, resulting in the attenuation of inflammation, whereas the other two pathways primarily work together to promote inflammation (Figure 7) (Djidjik et al., 2025).

### 6.5 Hypoxia-inducible Factor-1α signaling

The predominant features of sepsis include poor tissue perfusion, inability to deliver sufficient oxygen, and microvascular damage. These features lead to the stabilization and accumulation of HIF-1α in the nucleus (Tong et al., 2023). HIF-1α regulates several pathways, including glycolysis, angiogenesis, and cytokine production, among others (Ruan et al., 2024). NF-κB increases HIF-1α transcription, and several intermediates of metabolism and inflammation, such as succinate, inhibit the degradation of HIF-1α, creating a reciprocity between the regulation of inflammation and metabolism (Lotsios et al., 2024). HIF-1α is associated with the production of IL-1β, macrophage activation, glycolytic reprogramming and NLRP3 inflammasome activation, linking tissue hypoxia with innate immune signaling (Liu and Liang, 2025; Yuan et al., 2026).

### 6.6 Nrf2 antioxidant pathway

Nrf2 is widely described as the key transcription factor involved in the regulation of the expression of antioxidant genes (Liu et al., 2025). Under oxidative stress, Nrf2 is released from its complex with Keap1 and translocates into the nucleus, thus activating the expression of several antioxidant genes such as heme oxygenase-1 (HO-1), NAD(P)H quinone oxidoreductase 1 (NQO1), superoxide dismutase (SOD), catalase, and several glutathione enzymes (Kong et al., 2011; Yang X. R. et al., 2026). Nrf2 acts to decrease inflammation by modulating ROS, preventing the activation of NF-κB, preventing the assembly of the NLRP3 and preserving the function of mitochondria (Gunne et al., 2020). NF-κB and Nrf2 act to counter-regulate one another and determine the extent of injury by oxidative stress and the body’s antioxidant defense (Thimmulappa et al., 2006).

### 6.7 Programmed cell death pathways

Sepsis is categorized by unregulated interactions and dysregulation of both the innate immune system and programmed cell death pathways (Yang et al., 2023). Apoptosis is generally a non-inflammatory process; however, excessive lymphocyte apoptosis, along with dendritic cell apoptosis, contributes to the immune suppression that is characteristic of late-stage sepsis (Jiang and Cai, 2026). The inflammatory phase of apoptosis is mediated by caspase-1 and the human equivalents of caspase-4 and -5, and the mouse equivalent of caspase-11, which is activated by the NLRP3 (Luan et al., 2015). Pyroptotic cell death is characterized by the release of the pro-inflammatory cytokines IL-1β, IL-18, the protein HMGB1, ATP, and mtDNA, which further activate TLR, NF-κB, and the cGAS–STING signaling cascade (Wen et al., 2022). Necroptosis is mediated by a RIPK1, RIPK3, and MLKL protein complex (Du et al., 2022). Similar to pyroptosis, necroptosis is characterized by cellular rupture and the release of a large amount of DAMPs, which activate TLRs and cGAS–STING signaling pathways (Wang L. X. et al., 2025). Ferroptosis is driven by lipid peroxidation and contributes to mitochondrial dysfunction, oxidative stress and activation of the inflammasome and NF-κB pathways during sepsis (Zhao et al., 2025).

### 6.8 Complement system

The complement system interacts with and enhances TLR signaling as part of its role in innate immunity (de Nooijer et al., 2023). Complement components C3a and C5a are important for neutrophil recruitment and macrophage activation, and they increase blood vessel permeability and promote cytokine secretion (Yan and Gao, 2012). C5a enhances NF-κB and the NLRP3 and endorses oxidative stress (Markiewski et al., 2008; Khodabandeh et al., 2025). Complement activation in excess leads to organ damage and sepsis-related complications, including endothelial and blood clotting disorders.

### 6.9 Coagulation and immunothrombosis

The processes of inflammation and blood coagulation are closely linked in sepsis. Tissue factor expression, which activates the extrinsic pathway of blood coagulation, results from the activation of the TLR and NF-κB pathways (Ryan and O'Neill, 2022). Released from activated platelets, pro-inflammatory mediators augment TLR signaling and inflammasome activation (Maneta et al., 2023). Neutrophil extracellular traps (NETs) cause the activation of blood coagulation and also provide additional DAMPs that stimulate the cGAS–STING pathway and TLRs (Zhou K. et al., 2025). This interaction causes a pro-thrombotic state, DIC, and microclots in the blood and results in poor blood flow to the tissues (Aklilu et al., 2025).

### 6.10 Endoplasmic reticulum stress and the unfolded protein response

Unfolded protein response (UPR) is stimulated by the disrupted folding of proteins in the ER following stress, and disorganization in mitochondria-associated endoplasmic reticulum membrane (MAM) contributes much to the imbalance of innate immune response (Galluzzi et al., 2017). Damage associated with inflammation and ROS disrupts metabolism and leads to the UPR. There are three major sensors of ER stress: PERK, IRE1α, and ATF6 (Vivas and Weis, 2022). Chronic ER stress leads to further enhancement of NF-κB and NLRP3, increased damage to mitochondria, and activation of the cGAS–STING pathway (Khan et al., 2015). While inflammation and organ dysfunction become features of sepsis, severe ER stress leads to apoptosis and the formation of pro-inflammatory cytokines (Metzing et al., 2022; Gao L. et al., 2025).

### 6.11 Extracellular vesicles and neutrophil extracellular traps

Sepsis is associated with an overwhelming inflammatory response and dysregulated cell communication and signaling (Yan W. et al., 2025). Extracellular vesicles, such as exosomes, microvesicles, and apoptotic bodies, have been demonstrated to be important communicators during sepsis (Schiavello et al., 2023). Vesicles transfer and deliver cytokines, microRNAs, mtDNA, proteins, and lipids (Pfister, 2022). Extracellular vesicles drive the activation of the TLR-NF-κB-cGAS–STING axis and inflammasome signaling pathways within various tissues by the intercellular transfer of inflammatory molecules (You et al., 2025). Neutrophil extracellular traps (NETs), which are made of chromatin and antimicrobial proteins, are primarily intended to ensnare and kill pathogens, but also secrete extracellular DNA (Retter et al., 2025), which leads to cGAS–STING and TLR9 signaling and activates the inflammasome, producing an additional pro-inflammatory response and promoting thrombosis (Ortmann et al., 2024; Jiang et al., 2023).

### 6.12 Integrated signaling network and therapeutic implications

Sepsis progression involves multiple intersecting molecular pathways (Zhang and Ning, 2021). These include MAPK, PI3K/Akt/mTOR, AMPK, JAK–STAT, HIF-1α, Nrf2, coagulation and complement pathways, ER stress, programmed cell death, extracellular vesicles, and NET formation. These pathways are inextricably linked to TLR, NLRP3, cGAS–STING, and NF-κB signaling (Li Y. et al., 2025). Therapeutic efforts aimed at the single signaling pathway or cytokine level have proven to provide limited clinical outcomes (Wu H. et al., 2025). Instead, the focus has shifted to the manipulation of multiple therapeutic targets at once, that is, restoring mitochondrial activity, adjusting immunometabolism, promoting autophagy, decreasing oxidative stress, adjusting cell death pathways, maintaining the integrity of endothelial cells, and promoting balanced immune signaling networks (Saavedra-Torres et al., 2025). Future therapeutic strategies will likely include the manipulation of multiple therapeutic targets within this network, personalized concomitant therapeutic approaches and the integration of biomarker-guided therapeutic approaches (Duan et al., 2026). This type of therapeutic target manipulation is likely to preserve immune antimicrobial responses and limit the clinically evident inflammatory response in patients suffering from septic and other inflammatory conditions associated with dysregulated innate immune responses (Yilmaz et al., 2026; Liu and Liang, 2025).

## 7 Organ-specific injury in dysregulated innate immune signaling

### 7.1 Acute lung injury and ARDS

**FIGURE 8:**
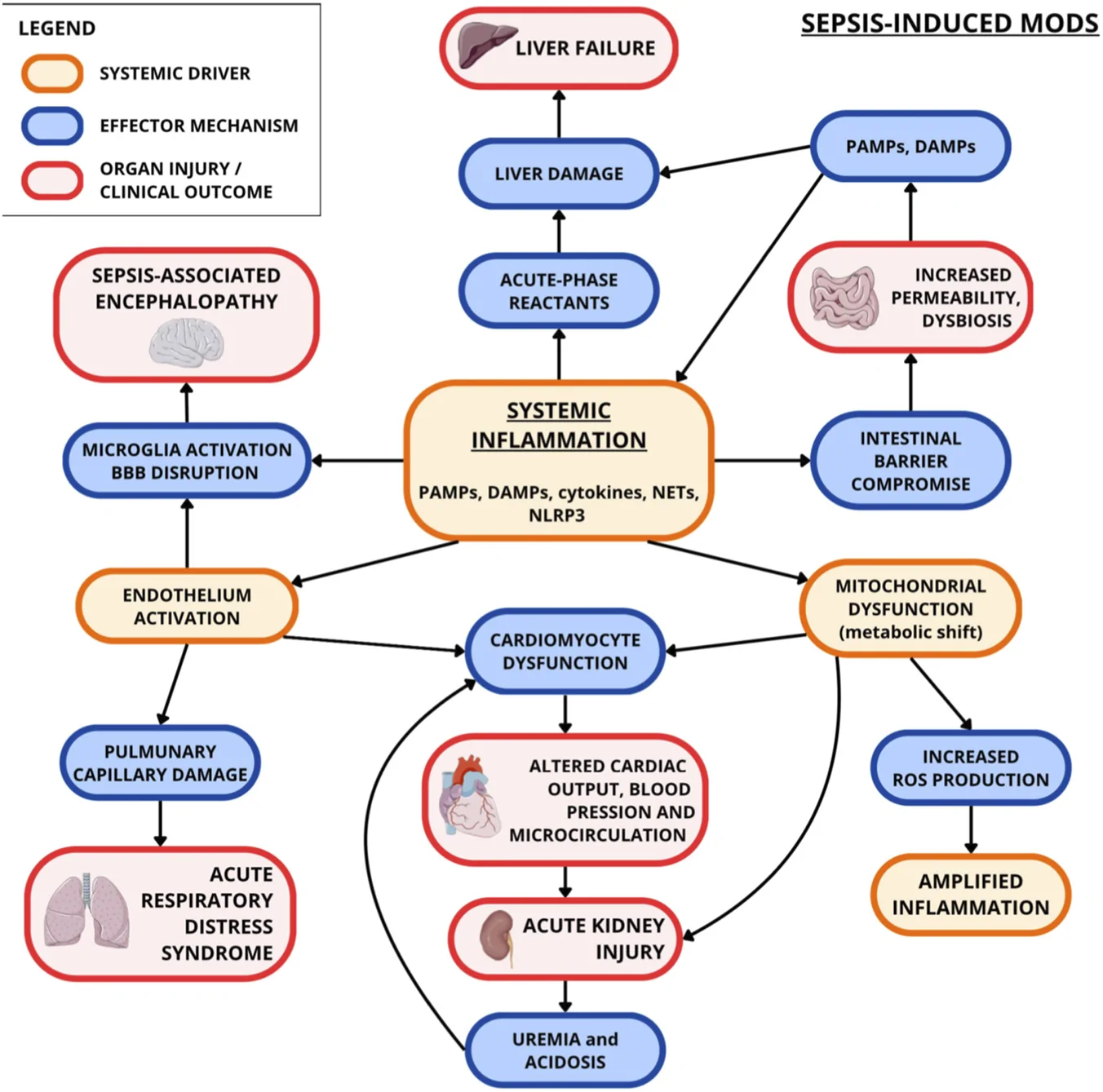
A schematic representation of the sepsis-induced multiorgan dysfunction syndrome (MODS). It is focused on the heart, kidneys, brain, liver, gut, and lungs, with an interplay of PAMPs, DAMPs, and cytokines, contributing towards the generation of extra cytokines and ROS, which further contribute to mitochondrial dysfunction and reduced energy metabolism. While inflammation is causing endothelial activation and damage. These two conditions display damaging effects on the respective tissues, resulting in dysfunction of the cardiomyocyte, kidney and liver, ARDS, dysbiosis, and encephalopathy (Vella et al., 2025). Reprinted from Front. Immunol., 1,682,306, Roberta V et al., “Cytokines in sepsis: a critical review of the literature on systemic inflammation and multiple organ dysfunction”, Copyright (2025), with permission from Frontiers.

Of all the organs, the lungs are affected early and severely in sepsis (Li B. et al., 2025). The presence of pathogens in the blood triggers inflammation in the lungs by activating macrophages and neutrophils in the air sacs and the lung epithelium and endothelium (Kumar, 2020b). TLR stimulation upregulates NF-κB, leading to the expression of TNF-α, IL-1β, IL-6, CXCL8, and other chemokines, recruiting neutrophils to the site of infection. Neutrophils damage the alveolar-capillary barrier with ROS and NETs (You et al., 2025). Damage to mitochondria and the generation of ROS activate NLRP3 and the cGAS-STING pathway (Li B. et al., 2025). Cleavage of macrophages by caspase-1 induces a form of cell death called pyroptosis, resulting in the release of effector molecules which further aggravate the inflammatory response in the lungs (Ma et al., 2025). The major pathological effects of this cascade are increased pulmonary barrier permeability, pulmonary edema, and the formation of hyaline membranes, leading to disrupted gas exchange, diffuse alveolar damage, and ARDS (Hu et al., 2020). In the later recovery stage of sepsis, persistent inflammation of the lungs eventually ends up in the development of pulmonary fibrosis (Figure 8) (Lu X. et al., 2025).

### 7.2 Acute kidney injury

Acute kidney injury (AKI) occurs quite frequently in septic patients and worsens outcomes (Liu et al., 2024; Pais et al., 2024). Multiple components result in renal injury, including microvascular hypoperfusion, endothelial and mitochondrial dysfunction, tubular epithelial inflammation, and immunothrombosis (Nian et al., 2025). Renal tubular epithelial cells have several pattern recognition receptors, including TLR2, TLR4, and TLR9, to detect invading pathogens and inflammation associated with tissue damage. NF-κB is activated upon recognition of stimuli, resulting in the formation of pro-inflammatory mediators that lead to the recruitment of macrophages and neutrophils (Zhao et al., 2020). Mitochondrial injury and release of DNA are coupled with the formation of ROS and promote the activation of the cGAS–STING pathway and NLRP3 inflammasome (Cao Y. et al., 2025). Pyroptosis, as a result of inflammasome activation, is the principal cause of cell death in tubular epithelial cells (Zarbock et al., 2023). Continuous activation of STING is a major cause of type I interferon-mediated inflammatory injury (Aguilar et al., 2024). A major characteristic of septic AKI is a combination of injury to tubular epithelial cells and endothelia, loss of cell polarity and of mitochondrial function, decreased glomerular filtration, and acute tubular necrosis (Zhao et al., 2023). Septic AKI is worsened by the failure of both autophagy and mitophagy (Yi et al., 2026).

### 7.3 Septic cardiomyopathy

Septic cardiomyopathy can develop in the majority of patients who present with severe sepsis and exhibits several features, including decreased cardiac output, increased arrhythmia, decreased ventricular contractility, and diastolic dysfunction (Boissier and Aissaoui, 2022; Zakynthinos et al., 2025). Cardiomyocytes have been shown to express TLRs and interact with systemic inflammation (Li W. et al., 2024). TLR4-NF-κB pathway activation leads to an inflammatory response and generation of nitric oxide and ROS, which creates an obstacle to excitation-contraction coupling (Fan and Wu, 2024). Mitochondrial dysfunction is perhaps the most relevant component in the development of septic cardiomyopathy, as the heart is the most energy-demanding organ (Chen X. S. et al., 2026). Excessive ROS, impaired mitochondrial function and oxygen utilization leading to ATP depletion and release of mitochondrial fragments activate the NLRP3 Inflammasome and the cGAS–STING pathway (Song et al., 2023). While inflammasome activation and subsequent pyroptosis lead to cardiomyocyte death, the STING pathway is directed toward eliciting a cardiac inflammatory response and an interferon response (Cao et al., 2026). Calcium dysregulation, endothelial dysfunction, microvascular thrombosis, and an imbalance of the autonomic nervous system and of energy substrate metabolism reconfigure the myocardium and amplify the effects of septic cardiomyopathy and contribute to heart failure (Lukic et al., 2024).

### 7.4 Liver dysfunction

The liver works as a metabolic organ and an immunological organ with functions comprising the clearance of pathogens, the synthesis of acute-phase proteins, and detoxification (Xu X. et al., 2025; Yan et al., 2014). Kupffer cells are the resident liver macrophages which express and secrete a variety of TLRs and respond rapidly to the presence of microbial products in the bloodstream (Cai Y. Z. et al., 2026). TLRs activate the NF-κB pathway, which results in the formation of various pro-inflammatory cytokines, including TNF-α, IL-6, IL-1β, and a diversity of chemokines (Beyer et al., 2022). Several insults to the liver result in the injury of hepatocytes, including oxidative stress, injury to the mitochondria and the microcirculation, and the activation of various inflammasomes (Hong et al., 2023). The cGAS-STING pathway, when activated, amplifies inflammation in the liver via the secretion of type I interferons (Xu X. et al., 2025). Cholestasis, hepatocellular injury, the inability to synthesize albumin and gluconeogenesis, and the presence of coagulation disorders and hyperbilirubinemia are the major pathological effects of hepatic inflammation (Guo et al., 2025). Disruption of the normal inflammatory signaling also alters the metabolism of bile acids, lipids and amines, and contributes to a variety of metabolic disorders (Geladari et al., 2025).

### 7.5 Sepsis-associated encephalopathy

Sepsis-associated encephalopathy (SAE) is a diffuse brain dysfunction that occurs when there is no infection of the CNS (Zhong H. et al., 2024; Gofton and Young, 2012). In the presence of a systemic inflammatory response, the BBB is compromised, and there is an influx of cytokines, immune cells, and DAMPs (Wang R. et al., 2024). Microglia are the CNS immune cells, and their activation is mediated by the TLR-NF-κB pathway (Li J. et al., 2025). Dysfunction of the neurons is also caused by the production of ROS, nitric oxide and excitatory neurotransmitters (Ito et al., 2022). Mitochondrial injury leads to the activation of the NLRP3-cGAS-STING axis and results in neuroinflammation, pyroptosis, and apoptosis of the neurons (Zhong H. et al., 2024). The major clinical symptoms of SAE are: delirium, altered level of consciousness, cognitive impairment, memory deficits and long-term neurocognitive dysfunction (Hong et al., 2023; Sonneville et al., 2023). Chronic neuroinflammation has also been the cause of cognitive dysfunction in sepsis survivors (Ito et al., 2022).

### 7.6 Endothelial dysfunction and vascular injury

Sepsis dysregulation of the innate immune system primarily targets the vascular endothelium (Martin-Loeches et al., 2025). Endothelial cells possess the ability to directly respond to inflammation via TLRs, cytokine receptors, and DNA-sensing pathways. With the activation of NF-κB, there is an upregulation of endothelial cell expression of ICAM-1, VCAM-1, E-selectin, tissue factor, cytokines and chemokines (Kattan et al., 2025). Inflammation further promotes leukocyte adhesion, platelet activation, coagulation, and vascular permeability (Tang et al., 2024). The activation of the NLRP3-cGAS–STING axis induces pyroptosis, intertwines oxidative stress and interferon signaling, and further impairs the endothelium (Fernandez-Sarmiento et al., 2022). The primary vascular complications of sepsis include: endothelial capillary leakage, hypotension, tissue edema, microvascular thrombi, DIC, and imbalance of perfusion. Endothelial dysfunction, therefore, links inflammation with organ failure during sepsis (McMullan et al., 2024).

### 7.7 Gastrointestinal barrier dysfunction

The gastrointestinal tract (GIT) plays a dual role during sepsis as a source and as a target of inflammatory mediator release (Zheng et al., 2026). Increased intestinal permeability is a consequence of the disruption of epithelial cell tight junctions by inflammatory cytokines and enables the translocation of bacteria, endotoxins and fungi to the circulation (Yan et al., 2024). Intestinal epithelial cells are capable of inflammation and cytokine production through the activation of NF-κB as a result of TLR stimulation by microbial products. The interplay between mitochondrial dysfunction and inflammasome activation disrupts the intestinal epithelial barrier, causing cell death (Cao et al., 2018). Changes in the gut microbiome toward dysbiosis are also a source of amplified inflammation by antagonizing microbial metabolites and by increasing the exposure to PAMPs (Yoseph et al., 2016). The compromise of the intestinal barrier elicits chronic inflammation on the systemic level due to the persistent stimulation of TLRs, NLRP3, cGAS–STING, and NF-κB (Liu et al., 2025).

### 7.8 Hematological and immune system dysfunction

Sepsis has the capacity to damage both the adaptive and innate immune systems (Zhang Y. et al., 2026). Neutrophils become overly activated and produce ROS at an increased rate, and also become resistant to normal apoptosis and produce NETs that play a key role in immunothrombosis (Pons et al., 2026). Macrophages and Monocytes become tolerant to endotoxins later on in the process, following a rapid initial immune response associated with the activation of NF-κB and the formation of pro-inflammatory cytokines (Herawati et al., 2024). This cytokine storm also drives apoptosis of lymphocytes and causes CD8^+^ T-cell and NK-cell dysfunction (Yamamoto et al., 2025). The dual stress of an increase in glucocorticoids and the cytokine storm leads to the dysfunction and apoptosis of B-cells (Delano and Ward, 2016). The combination of persistent cGAS–STING signaling and chronic type I interferon production results in an exhausted immune system that becomes susceptible to secondary infections (Cao et al., 2023).

### 7.9 Skeletal muscle dysfunction and metabolic failure

Acute skeletal muscle wasting is a prevalent complication of sepsis in critically ill patients (Yoshihara et al., 2023). Sepsis-induced skeletal muscle injury is the result of an inflammatory response at the level of the muscle fiber where the cytokines activate NF-κB (Ono et al., 2026). This response results in the upregulation of muscle-specific E3 ubiquitin ligases MuRF1 and Atrogin-1 that increase muscle proteolysis (Wu Q. et al., 2025). Sepsis also induces a worsening of oxidative stress and an inhibition of mitochondrial ATP production, which greatly damages the contractile and regenerative capacity of muscle (Zolfaghari et al., 2015; Kubat et al., 2025). Sepsis-induced inflammation and stress cause a further worsening of insulin and metabolic dysfunction (Pierre et al., 2025). Collectively, these factors greatly contribute to the long-lasting disability linked with sepsis (Mankowski et al., 2021).

### 7.10 Multiple organ dysfunction syndrome

MODS is associated with the extreme effects of unregulated innate immune responses that occur in sepsis, and it is not due to the effects of a few organ-specific responses (Wu et al., 2026). It is a consequence of the integrated, systemic, inflammatory response networks that involve TLRs, the NLRP3 inflammasome, cGAS–STING, NF-κB, complement, immunothrombosis, oxidative stress, and endothelial and metabolic reprogramming, as well as dysregulation of programmed cell death (Gando, 2010; Maiden and Sasson, 2026). Pathway crosstalk and persistent stimulation reinforce inflammatory circuits that result in excess cytokine production, endothelial cell injury, mitochondrial dysfunction, coagulopathy, and global hypoxia (Wang and Ma, 2008; Mun et al., 2025). In addition to the effects of unrestrained inflammation, there is the development of compensatory forms of immune system depression that lead to endotoxin tolerance, lymphocyte death, and the exhaustion of immune and metabolic functions (Gourd and Nikitas, 2020). These sustain the risk of persistent and opportunistic infections. Because these pathogenic mechanisms are shared across multiple organs, MODS is better defined as loss of the integrated systemic inflammatory response (Bhatia, 2021). Thus, if therapeutic measures restore mitochondrial function, allow the endothelium to maintain its barrier functions, and diminish the effects of oxidative stress, as well as autophagy and TLR–NLRP3–cGAS–STING–NF-κB signaling, they provide a better framework to protect multiple organs from the effects of MODS (Zhong et al., 2026). From a mechanistic standpoint, the precision of approaches to treat unrestrained inflammation and restore dysregulated innate immunity is crucial to prevent the transitory effects of unrestrained inflammation, which ultimately lead to MODS (Tang et al., 2024).

## 8 Therapeutic targeting of innate immune networks

### 8.1 TLRs signaling

TLRs are considered valuable targets for the development of new therapies, possibly due to their role in initiating the innate immune response. (i) TLR4 Antagonists. As a receptor for bacterial LPS, TLR4 is one of the first receptors to be engaged in signaling during Gram-negative sepsis (Kanzler et al., 2007). Several TLR4 antagonists have been shown to have beneficial effects in experimental sepsis by decreasing NF-κB activation, cytokine production, impairment of the endothelium, and injury to the organs (Shin et al., 2025). However, the clinical outcomes of these studies have been underwhelming and instructive, as broad inhibition of TLR4 may lead to a complete failure to clear the infecting organisms (Cao et al., 2022). (ii) MyD88 and TRIF Modulation (Zhu and Mohan, 2010). The inhibition of signaling by the downstream TRIF and MyD88 proteins has been shown to decrease the inflammatory response, while still allowing some protective innate immune responses to be maintained (Lawton and Ghosh, 2003). Inhibition of MyD88 signaling has been shown to decrease cytokine production in experimental studies (Farooq et al., 2021). (iii) TLR9 Inhibition. Due to the activation of TLR9 by mtDNA from dying cells during sepsis, TLR9 selective antagonists are being explored as therapies for sterile inflammation and organ injury (El-Zayat et al., 2019). More generally, the most promising therapeutic strategy is TLR signal modulation (Uematsu et al., 2004).

### 8.2 NLRP3 inflammasome

Of various inflammasome targets, the NLRP3 inflammasome is the most promising target since it has the potential to amplify inflammation caused by a myriad of signaling pathways ((Zhou et al., 2026; El-Sharkawy et al., 2020; Ma, 2023; Prakash et al., 2023). (i) Direct NLRP3 Inhibitors. Although a few small-molecule NLRP3 inhibitors (e.g., MCC950) have entered clinical studies (Jiang et al., 2020), the other NLRP3 inhibitors, OLT1177 (dapansutrile) (Sanchez-Fernandez et al., 2019), CY-09 (Fan et al., 2021), and Tranilast (Saeedi-Boroujeni et al., 2022), have been shown to target the NLRP3 ATPase, NACHT domain, and inhibit inflammasome assembly, respectively (Li et al., 2026; Jiang et al., 2017; Matico et al., 2025; Seok et al., 2021; Zhen et al., 2026). These small molecules significantly decrease experimental sepsis organ injury, endothelial dysfunction, IL-1β, pyroptosis, and sepsis-induced inflammation (Zhong H. et al., 2024). (ii) Caspase-1 Inhibitors. Caspase-1 inhibitors have the potential to decrease the maturation of both IL-1β and IL-18, as well as gasdermin D-mediated pyroptosis (Ustundag, 2026; McKenzie et al., 2018; Wei et al., 2022; Duan et al., 2024). (iii) Gasdermin D Inhibitors. Since pyroptosis induces tissue injury, gasdermin D inhibition may be one of the few therapeutic strategies to decrease inflammatory cell death while still preserving the ability of the immune system to recognize and respond to injury (Liao et al., 2026; Dai et al., 2023).

### 8.3 cGAS–STING signaling

The cGAS–STING signaling cascade is an emerging pathway of sterile inflammation and mtDNA-mediated immune activation (Huang et al., 2023; Mahajan et al., 2026). (i) cGAS Inhibitors. cGAS inhibitors prevent the synthesis of cGAMP and the associated inflammatory response (Li Q. et al., 2021; Xiao et al., 2025). (ii) STING Antagonists. Some exploratory compounds inhibit STING by preventing the LTING domain conformational changes and intracellular transport (Luo et al., 2025). These compounds may have a beneficial therapeutic effect by decreasing type I interferons and NF-κB-associated endothelial injury and improving mitochondrial function (Mahajan et al., 2026; Liu H. et al., 2026). (iii) TBK1 Inhibitors. Since TBK1 is located downstream of STING, selective TBK1 inhibition results in the blockade of both interferon and inflammatory cytokines (Zhang et al., 2022; Jiang L. et al., 2026). Due to the important host defense roles of cGAS-STING, careful selection of patients and timing of therapies are warranted (Cai et al., 2025).

### 8.4 Targeting NF-κB pathway

NF-κB occupies a central control point of all innate immune responses and is therefore an attractive drug development target (Iacobazzi et al., 2023; Yu et al., 2020). These include IKK inhibitors, blockers of the translocation of NF-κB into the nucleus, proteasome and antioxidant inhibitors, glucocorticoids, and phytochemicals (Fu et al., 2026). Natural products such as curcumin, resveratrol, quercetin, berberine and epigallocatechin gallate (EGCG) have all been shown to block NF-κB activation with a concomitant decrease in oxidative stress and inflammasome activation (Chauhan et al., 2022; Guo et al., 2024; Markopoulos et al., 2025). NF-κB also has a key role in the regulation of immunity to infections and cell death (Bakrim et al., 2025). Hence, the goal is to eliminate NF-κB activity and to selectively modify NF-κB activity depending on the stage of the disease, the immune system, and the phenotype (Herrington et al., 2016; Khasanov et al., 2026).

### 8.5 Targeting immunometabolism

The recognition of the role of metabolic reprogramming of immune cells has created new avenues for anti-inflammatory therapies (Kumar, 2020a; Shin et al., 2025). (i) AMPK Activators. AMPK activation promotes the following: Mitophagy, Biogenesis of mitochondria, Fatty acid oxidation, and Autophagy (Ulevitch, 2004; Huang et al., 2024). In parallel, AMPK suppresses NF-κB activation, NLRP3, mitochondrial ROS, and cGAS-STING (Day et al., 2017; Prantner et al., 2017; Jo et al., 2019). (ii) Metabolic Reprogramming. Therapies targeting the metabolism of the following (Deng et al., 2025; Mohammadnezhad et al., 2022): glycolysis, glutaminolysis, succinate metabolism, and itaconate pathways have provided significant alterations of macrophage metabolism and polarization, providing strong anti-inflammatory responses (Mulder et al., 2019; Palsson-McDermott and O'Neill, 2020; Ning et al., 2023). (iii) mTOR Modulation. Discerning inhibition of mTOR will endorse autophagy and improve mitochondrial quality control, with reductions in inflammatory signaling (Weichhart et al., 2015; Saemann et al., 2009; Jiang X. et al., 2026).

### 8.6 Mitochondrial protection and antioxidant therapy

Since there are multiple inflammatory pathways that are triggered by mitochondrial dysfunction, protecting the mitochondria has become a priority for many therapies (Zhong et al., 2026). These include: (i) Mitochondrial-targeted antioxidants. MitoQ, SkQ1, and SS-31 (elamipretide) diminish oxidative stress in the mitochondria, enhance the production of ATP, and inhibit the activation of the NLRP3 and cGAS–STING pathways (Fock and Parnova, 2021; Li et al., 2013). (ii) Nrf2 activators. Nrf2 pathway activation promotes antioxidant responses through the upregulation of heme oxygenase-1, NQO1, and glutathione-related enzymes, which leads to a reduction of ROS as well as the indirect inhibition of NF-κB and inflammasome activation (Dinkova-Kostova and Copple, 2023; Pant et al., 2024; Manful et al., 2025). (iii) Mitophagy enhancers. It has been shown that the elimination of defective mitochondria through the pharmacological induction of mitophagy prevents the release of ROS and mtDNA, and subsequently disrupts several inflammatory signaling pathways that are triggered by the former (Song et al., 2020; Gkikas et al., 2018; Cho et al., 2020).

### 8.7 Biologic therapies and cytokine modulation

Several biologic therapies focused on the modulation of inflammatory cytokines have been studied in septic patients, such as IL-1 receptor antagonism, anti-IL-6 monoclonal antibodies, TNF-α inhibition, GM-CSF modulation, and interferon therapies (Vella et al., 2025; Mulder et al., 2019; Schulte et al., 2013). While some therapies that focus on cytokine modulation have shown some positive effects in some patient populations, the general restriction of cytokines has resulted in an adverse effect on the ability to fight infections (Shin et al., 2025; Xu Y. et al., 2025). It is expected that with the use of biomarkers and other personal immune system assessments, future biologic therapies will target selected patients for precision medicine (Yang Y. et al., 2026; Liang et al., 2025).

### 8.8 Epigenetic and RNA-Based therapeutics

Modulating epigenetic changes for therapeutic interventions exemplifies a novel treatment approach (Ivashkiv, 2018). This includes the use of histone deacetylase (HDAC) inhibitors, DNA methyltransferase inhibitors, bromodomain (BET) inhibitors, and agents that modulate histone acetylation (Dai et al., 2024). RNA therapeutics that target lncRNAs and miRNAs have the potential to modulate many cellular pathways and processes concurrently (Zhang et al., 2020c; Gareev et al., 2023). For example, enhancement of miR-146a diminishes TLR–NF-κB pathway activation, delivery of miR-223 antagonizes the NLRP3 inflammasome, and modulation of miR-155 affects macrophage polarization and alterations in the synthesis of pro-inflammatory cytokines (Xie et al., 2026; Di Martino et al., 2021; Pandey and Yadav, 2025). Due to the capacity of these therapeutic agents to modulate signaling networks at the system level, their therapeutic potential is vast (Cao et al., 2022).

### 8.9 Stem cell and extracellular vesicle therapies

Mesenchymal stem cells (MSCs) have demonstrated remarkable therapeutic effects in experimental sepsis, likely due to their immunomodulatory effects (Martin-Rufino et al., 2019; Akbar et al., 2021; Farm et al., 2025). MSCs promote the suppression of overwhelming inflammatory responses, enhance the clearance of bacteria, promote the healing of damaged tissues and restoration of the endothelium and improve the mitochondrial functioning (Burrello et al., 2016; Lee et al., 2026). MSC-derived extracellular vesicles (EVs) provide additional treatment options to modulate inflammation and improve the function of the endothelium and the mitochondria, without the limitations associated with MSCs (Gomzikova et al., 2019; Jouybari et al., 2024). MSC-derived EVs carry anti-inflammatory miRNAs and other important proteins and factors, suggesting that the use of EVs to modify the effects of inflammation will be a positive therapeutic approach for the treatment of sepsis (Kumar et al., 2024; Liu H. et al., 2021).

### 8.10 Nanomedicine and targeted drug delivery

TLR inhibitors, NLRP3 inhibitors, STING pathway inhibitors, small interfering RNAs (siRNAs), antioxidants, and anti-inflammatory agents are examples of substances that are being explored to determine if they can be delivered to specific tissues using novel drug delivery systems (Zhao et al., 2025; Lian et al., 2026; Pednekar et al., 2024). Nanotechnology can be employed to refine therapeutics and minimize systemic toxicity (Ci et al., 2024; Dhar et al., 2026). The technology allows for targeted delivery and accumulation of therapeutics to tissues with inflammatory responses (Li et al., 2025a). The use of nanotechnology to develop therapeutics that are responsive to specific inflammatory conditions to modulate inflammation and oxidative damage is a promising approach for the treatment of sepsis (Ramavat et al., 2025; Croitoru et al., 2024). Recently, one development has been documented in the therapeutics of inflammation based on the cytokine storm (You, 2025). Nanoparticles have been developed, integrated with antibiotic and anti-inflammatory molecules, that is, tannic acid-Mn^2+^-polymyxin-B PVP nanoparticles (TMPPs) (Yim et al., 2020). Polymyxin B is bactericidal, neutralizes LPS, disrupts TLR-4 regulated signaling and suppresses the synthesis of proinflammatory cytokines. Tannic acid scavenges ROS and neutralizes cell-free DNA, both of which are inducers of cytokine storm. This explains the therapeutic potential of nanomedicine in the modulation of hyperinflammatory responses.

### 8.11 Biomarker-guided precision medicine

Sepsis is an incredibly heterogeneous disorder, signaling the need for more personalized treatment methods (Ulevitch, 2004). Some novel biomarkers are: circulating mitochondrial DNA, IL-1β, IL-6, procalcitonin, HMGB1, cell-free DNA, soluble TREM-1 (soluble triggering receptor expressed on myeloid cells-1), transcriptomic immune signatures, and metabolomic profiles (Padovani and Yin, 2024; Lopez-Cruz et al., 2024; Cai Y. Z. et al., 2026; Mulder et al., 2019). The use and integration of multi-omics and other related fields, up to single-cell resolution, can identify patient-specific immune phenotypes (Duan et al., 2026; Shin et al., 2025). The use of AI and predictive ML techniques can help guide individualized immune system response therapeutics, disease progression, and response to treatment prediction (Sun et al., 2025; Jiang W. et al., 2026).

### 8.12 Combination therapies targeting integrated networks

It is becoming clear that no single/signaling pathway can be used to explain the pathogenesis of sepsis (Wang Y. et al., 2026). Sepsis is characterized by the convergence of TLR, NLRP3 inflammasome, cGAS–STING, NF-κB, immunometabolic, mitochondrial, and vascular signaling pathways (Zhang Y. et al., 2026; Ulevitch, 2004). The next-generation of sepsis therapies will likely be based on the principles of combination therapy (Lopez-Cruz et al., 2024). This will include the integration of early directed antimicrobial therapy and source control with immunomodulation, mitochondrial therapy, autophagy and mitophagy, antioxidants, endothelial therapy, immunometabolic reprogramming, and biologic therapy, guided by inflammation biomarkers (Padovani and Yin, 2024). The goal of these combination therapies will be to inhibit sepsis-related pathology and dysregulation in the immune response while promoting repair of injured tissues (Ovali and Percin, 2025; Shin et al., 2025).

## 9 Emerging technologies for studying molecular crosstalk

### 9.1 Single-cell transcriptomics

The ability to analyze and describe the molecular makeup of individual cells through single-cell omics technologies is one of the most significant innovations in immunology (Baek and Lee, 2020; De Rop et al., 2024). (i) Single-cell RNA sequencing (scRNA-seq). The main value of single-cell RNA sequencing is its ability to provide transcriptomic data on thousands of individual cells at once (Tarozzi et al., 2026). This capability allows distinction among various subtypes of immune cells, their activation states, and the signaling pathways they engage during sepsis (Kanter and Kalisky, 2015). Some of the many potential applications include the identification and characterization of activated macrophage subsets, neutrophil heterogeneity, and immune exhaustion (Kulkarni et al., 2019). Additionally, it allows the analysis of pathway activation during sepsis involving TLR, NLRP3, cGAS–STING and NF-κB, and the temporal monitoring of immune responses (Nayak and Hasija, 2021; Song et al., 2019). scRNA-seq has uncovered novel immune cell populations which participate in sepsis-related inflammation, immune suppression, and other aspects of immune dysregulation. (ii) Single-cell ATAC sequencing (scATAC-seq) (Wang C. et al., 2025). scATAC-seq evaluates accessible chromatin, thereby allowing the identification of regulatory elements involved in the transcription of inflammatory genes (Shi et al., 2022; Chen et al., 2019). When scRNA-seq and scATAC-seq are combinatorially applied, they elucidate the role of various transcription factors, including NF-κB, IRF3, members of the STAT family, AP-1, and HIF-1α (Xiong et al., 2019). (iii) Single-cell multi-omics. From a single cell, it is possible to perform simultaneous transcriptomic, epigenomic, proteomic, metabolomic, and chromatin accessibility analyses, which permit the elucidation of extensive signaling pathways (Pan et al., 2024; Lee et al., 2020; Liang et al., 2024; Wu X. et al., 2024).

### 9.2 Spatial omics

While single-cell sequencing allows the documentation of cellular heterogeneity, it often lacks information on the spatial distribution of cells and their interactions within a tissue (Lee et al., 2025). Spatial omics resolves this limitation and allows measurement of gene or protein expression while maintaining the architecture of the tissue (Li X. et al., 2025). (i) Spatial transcriptomics. This approach provides a means to measure gene expression within a tissue section and allows the visualization of sepsis-related inflammatory signaling pathways in the different organ systems (Bernardini et al., 2026). Some of the many possible applications include the following (Wu M. et al., 2024): localization of activated macrophages; mapping of endothelial cell inflammation; identification of inflammation-associated microenvironments; analysis of cytokine gradients; and the characterization of immune responses in different organs (Christopher et al., 2022; Zhou X. M. M. et al., 2025). (ii) Spatial proteomics. Imaging technologies are advancing to a stage where dozens of proteins may be mapped to tissue samples *in situ* (Xiang et al., 2023). Pioneering work in imaging techniques permits the visualization of TLR4, NLRP3, STING, phosphorylated NF-κB, and numerous cytokines (Mao et al., 2021). It has been especially useful in elucidating the dynamics of immune cell interactions in the context of organ injury (Piyadasa et al., 2023).

### 9.3 Multi-omics integration

Protein function is mainly dictated by PTMs rather than gene expression (Baiao et al., 2025). Mass spectrometry-based proteomics offers the possibility of studying the relative abundance of proteins, protein interactions, and PTMs, including phosphorylation, ubiquitination, acetylation, SUMOylation, and glycosylation (Subramanian et al., 2020). Phosphoproteomics is of particular value since the activation of TLR, cGAS–STING, MAPK, and NF-κB is primarily dependent on phosphorylation (Tong et al., 2025). These techniques allow the mapping of signaling pathways and the discovery of new regulatory proteins (Duan et al., 2024; Qian S. et al., 2026). Metabolomics aids in profiling the myriad metabolites that influence the activation of the innate immune system (Sibilio et al., 2025). The pathways of interest are primarily related to glycolysis, TCA cycle, amino acid metabolism, fatty acid oxidation, and lipid metabolism (Duan et al., 2026). It has been shown that succinate, itaconate, citrate, lactate, and fumarate modulate the activation of NF-κB, the NLRP3, the polarization of macrophages, and the cGAS–STING pathway (Liu F. et al., 2026; Wan and Wang, 2025). The coupling of metabolomics with transcriptomics and proteomics helps to elaborate the mechanisms of immunometabolic processes in sepsis (Nguyen et al., 2025).

### 9.4 Functional genomics and high-resolution imaging

The advent of CRISPR genome editing technologies has had an enormous impact on the field of functional immunology (Jia et al., 2024; Sinha et al., 2018). (i) Genome-wide CRISPR libraries help identify genes that control components of TLR regulation, inflammasome activation, STING signaling, cytokine secretion, and immunometabolism (Wu M. et al., 2020). CRISPR activation (CRISPRa) and interference (CRISPRi) provide tools for the modulation of gene expression through the activation and silencing of individual genes, respectively (Allemailem et al., 2023). These tools allow for targeted analysis of the interplay of different signaling pathways with minimal off-target effects (Allemailem, 2024). (ii) Base editing and prime editing. Targeted genome editing technologies allow for the correction of mutations with therapeutic value (Li et al., 2025b). These technologies may allow for highly precise assessment of mutations for the advancement of personalized medicine in the future (Antoniou et al., 2021; Nallajennugari et al., 2026).

Advanced imaging technologies make it possible to visualize the signaling events in real time. Key technologies consist of: (i) Super-resolution microscopy. The assembly of inflammasomes, speck formation by ASC (apoptosis-associated speck-like protein containing a CARD), trafficking of STING, translocation of NF-κB, and the dynamics of mitochondria are examples of what super-resolution imaging can allow us to visualize (Kubalova et al., 2021). (ii) Live-cell imaging. Signaling highly dynamic events, such as the signaling of calcium, the generation of ROS, autophagy, pyroptosis, and cell migration, can be captured in real time by this imaging technology (Nallajennugari et al., 2026; Garcia-Gimenez et al., 2026). (iii) Intravital microscopy. Intravital microscopy makes it possible to observe the behavior of immune cells, injury of blood vessels, and inflammatory signaling in the real-time development of experimental sepsis (Sumen et al., 2004; Wang J. et al., 2024).

### 9.5 Systems biology and network medicine

Since signaling for the innate immune system consists of many thousands of interacting components, systems biology is necessary (Fernandez-Sarmiento et al., 2022). Integration of gene regulatory networks, protein interaction networks, metabolic pathways, signaling pathways and cellular interactions is termed network biology (Addissouky et al., 2023). The effects of perturbation of a signaling pathway on a network of interrelated pathways are assessed by systems biology (Sinha et al., 2018). It has been shown within network medicine that more therapeutic value may be gained from the modulation of several signaling pathways compared to targeting single cytokines (Pierre et al., 2024).

### 9.6 Artificial intelligence and network modeling

The analysis of multi-omics datasets has become significantly easier with the advent of AI technologies (Bignami et al., 2025). ML provides the potential to discover new biomarkers, stratify and classify patients, predict the severity of a disease, locate new targets for interventions, repurpose drugs, and design and construct interactive signaling pathways (Qiao and Cui, 2022; Shanmugam et al., 2025). In addition, the potential of deep learning (DL) methodologies to assimilate datasets of varying modalities, such as transcripts, proteins, metabolites, images, and other clinical datasets, has the potential to expose novel regulatory relationships of TLR, NLRP3, cGAS-STING, and NF-κB in the immune system (Shen et al., 2026; Jin et al., 2025). Explainable AI becomes essential to model the complexities of molecular signaling networks and augment clinical decisions (Li F. et al., 2024).

### 9.7 Digital twin approaches for sepsis

Digital twin technologies are grounded in individual mathematical models that are built through a computational framework of patient-specific clinical and omics data (Danesh et al., 2024). Digital twins encompass clinical data, multi-omics data, imaging, immune phenotyping, and biomarker data collected over time. Personalized models within this framework may ultimately have the ability to predict disease, optimize treatment, and respond to drugs in a way that provides individualized immunomodulatory therapies for patients suffering from sepsis (Wang B. et al., 2026). Digital twin technologies have the potential, even in their nascent stages, to revolutionize the field of precision critical care medicine.

### 9.8 Organoid and organ-on-a-chip technologies

The traditional methods of cell culture typically cannot reproduce the complex microenvironments of tissues in sepsis (Moore et al., 2025). (i) Organoids. The capacity of 3D organoids to model *in vitro* tissue-specific and organ-specific inflammatory pathways is unparalleled (Wang L. X. et al., 2025). Organ-specific and inflammatory pathway-specific organoids have been developed for lung, liver, kidney, brain, intestine, and heart (Wu L. et al., 2023). These organoids enable a multitude of inflammatory pathway signal research relevant to TLR, inflammasomes, and STING pathways (Li et al., 2025c). (ii) Organ-on-a-chip. Microfluidic technologies have the capacity to model the mechanical forces of flow and immune cells and disrupt the endothelial cell barrier to study the effects of diffusing cytokines (Singh et al., 2022; Silva et al., 2026). These systems can be used to study the effects caused by drugs and how they interact with multiple organs (Pierre et al., 2024). There are several multi-organ chip systems available that can mimic the effects of systemic inflammatory response caused by sepsis (Kong et al., 2026).

### 9.9 Integration of multiple omics

The integration of multiple omics technologies stands as one of the greatest advances in modern immunology (Song et al., 2019; Qiao and Cui, 2022). Integrated analysis refers to the combination of genomics, epigenomics, transcriptomics, proteomics, phosphoproteomics, metabolomics, lipidomics, and microbiomics (Zhang Y. et al., 2026; Duan et al., 2026; Sibilio et al., 2025). Advanced computation in this domain can construct a systems-level understanding of signaling crosstalk and may help uncover key regulatory nodes in the context of disease (Wu X. et al., 2024; Wan and Wang, 2025). The integration of multiple omics is particularly powerful for the discovery of intricate diagnostic biomarkers and agile therapeutic targets (Liang et al., 2024; Qian S. et al., 2026).

## 10 Challenges and future perspectives

### 10.1 Complexity of innate immune signaling

An example of an innate immune system signal response is the interaction of several pathways, including TLRs, NLRP3 inflammasomes, cGAS-STING, NF-κB, MAPK and others (Zhang Y. et al., 2026). Each of these pathways signals components and receptors, and all involve feedback and complexity, while one signaling pathway will often initiate several other pathways (Shin et al., 2025). Thus, the blockage of therapeutic cytokines/signaling pathways will rarely have a positive effect (Cao et al., 2022). Rather than aiming to isolate the blockers of mediators, research should aim to identify the regulators of inflammatory pathways (Cao et al., 2022).

### 10.2 Dynamic nature of immune responses

The innate immune response to sepsis is best described as a rapidly changing response in which the immune system evolves to counteract the effects of the persistent infection (Lu X. et al., 2025). The sepsis continuum describes the following phases of immune response to sepsis: early hyperinflammation, immune cell dysregulation associated with inflammation, immune cell dysregulation associated with depression, and persistent immune cell dysregulation associated with chronic critical illness (Saavedra-Torres et al., 2025). During the continuum of sepsis, the activation of various signaling pathways leads to severe negative effects of implementing some therapies at certain phases of the continuum of sepsis, while other therapies may be beneficial (Yilmaz et al., 2026).

### 10.3 Patient heterogeneity and precision medicine

Sepsis includes a diverse range of patient populations (Liang et al., 2024). Variance arises from differences in infectious agents, infection sources, host genetics, age, sex, prior health conditions, immune system status, microbiome composition, and other environmental factors (Rio et al., 2025). These variances influence TLR, NLRP3, cGAS–STING, and NF-κB pathway signaling (Saavedra-Torres et al., 2025; Qian S. et al., 2026). The future of clinical treatment will involve individualized immune profiling, and it is possible that precision medicine will be based on specific biomarkers and will allow the tailoring of pathway-focused immunomodulatory treatments (Hossan et al., 2026; Zhang G. et al., 2026).

### 10.4 Limitations of experimental models

Animal models have been important for advances in sepsis research, but they cannot capture the complexity of the human disease (Dardalas et al., 2019). Limitations of models include: young, genetically identical individuals, pathogen exposure limited to one, no chronic disease, limited immune response complexity, and short evaluation periods (Nallajennugari et al., 2026; Rio et al., 2025). There are substantial differences in immune response, inflammatory responses, and metabolic responses between animal models and humans (Aydın and Bekmez, 2023). Additionally, differences in microbiomes and immune systems are substantial. Future research should incorporate more of the following: human organoids, organ-on-chip technologies, humanized models, *ex vivo* patient-derived tissues, and clinical single-cell analysis research (Li Y. et al., 2025; Mai et al., 2012). These methodologies may be more successful for translational research and testing of innovative therapies. However, there is a lack of robust biomarkers to accurately report on the status of the innate immune system response (Lopez-Cruz et al., 2024). Existing biomarkers such as procalcitonin, C-reactive protein, IL-6, and lactate only indicate partial disease progression and the status of the immune system (Padovani and Yin, 2024). Future development of biomarkers should consider circulating mitochondrial and cell-free DNA, inflammasome components and cytokines, as well as transcripts, proteins, metabolites, epigenetic modifications, and extracellular vesicles (Ovali and Percin, 2025; Xie et al., 2026). There may be potential to improve the early detection, prognosis, and even the monitoring of therapies, as well as the prognosis of the repercussions of multiple biomarker integration (Wang Y. et al., 2026).

### 10.5 Challenges in therapeutic development and future perspectives

Numerous agents modulating the immune system have exhibited great potential in laboratory models but later failed in human clinical trials (Duan et al., 2024; Renfro et al., 2016). Some of the reasons for these failures may include: delayed treatment initiation, insufficient biomarker-driven treatment, selection of the wrong patients for the trial, focus on the blockade of a single inflammatory mediator, ignoring the changing equilibrium of the immune system, adverse effects of the treatment, and maintaining the balance of the immune system (Luo et al., 2025; Dai et al., 2024). The design of future clinical trials should be flexible and incorporate real-time immune system monitoring, treatment response, and molecular biomarkers (He et al., 2024). Further, a great opportunity for the future also lies in integrating diverse molecular datasets. The emerging concept of the signals from TLR, NLRP3 inflammasome, cGAS–STING, and NF-κB pathways appear to be integrated within a complex system of innate immunity and needs to be explored further. The combination of single-cell and spatial multi-omics, AI, and advanced experimental systems should create an intricate map for novel targets for therapeutic interventions and robust biomarkers. Clinically, combinations of therapies should focus on mitochondrial dysfunction, oxidative stress, immunometabolic dysregulation, and autophagy. Overall, a collaborative approach between immunologists, intensivists, systems biologists, engineers, and pharmacologists will improve the treatment of sepsis from care-based management to systematic and advanced treatments that improve patient survival, length of time and quality of life after discharge.

From a clinical perspective, the translation of mechanistic insights into effective sepsis therapies remains a formidable challenge. Despite robust preclinical evidence supporting TLR4 antagonists, NLRP3 inhibitors, and STING antagonists, clinical trials have consistently failed to demonstrate mortality benefit when applied to unselected patient populations. This discrepancy underscores the need for biomarker-guided patient selection and immunophenotyping to identify subgroups most likely to benefit from pathway-targeted interventions. For instance, patients with persistently elevated plasma mtDNA or cGAS-STING activation signatures may represent a targetable endotype suitable for cGAS/STING inhibitor trials. Similarly, patients demonstrating early NLRP3-driven hyperinflammation may be candidates for MCC950 or analogous compounds. Future clinical trials should incorporate adaptive enrichment designs, Bayesian platforms, and validated inflammatory biomarker panels to allow dynamic patient selection. The integration of digital twin modeling and AI-driven clinical decision support further holds promise for real-time immunomonitoring and personalized therapeutic adjustment.

## 11 Conclusion

Despite improvements in antimicrobial therapies and intensive and supportive treatment, sepsis is still one of the most common causes of death globally, and it still causes considerable clinical and socioeconomic challenges. It is known that sepsis is the result of a disordered continuum of innate immune systems engaged in the signaling of infection, inflammation, immune system regulation, cellular metabolism, and death, in addition to the destruction and repair of tissues. TLRs, the NLRP3 inflammasome, the cGAMP–STING pathway, and NF-κB all provide the molecular basis of the signaling systems in sepsis. The dysregulation of the stimulatory and regulatory aspects of the immune system results in a cytokine storm, death of tissues, dysfunction and death of mitochondria, and inflammation of an injury to all the organs. Therapeutic interventions for sepsis should be aimed at the modulation of this dynamic and diverse signaling system as opposed to the activity of individual signaling systems. In particular, the negative regulation of the TLR, NLRP3, cGAS, and NF-κB signaling systems should be the focus of therapeutic interventions. The combination of these approaches will significantly benefit the treatment of sepsis. In addition to the improvement of diagnosis and treatment of sepsis, the combination of precision medicine, advanced molecular technologies, and biomarker-guided patient stratification will offer the opportunity to improve the prognosis of sepsis.

## Glossary

AKI: Acute kidney injury
AP-1: Activator protein-1
ARDS: Acute respiratory distress syndrome
ASC: Apoptosis-associated speck-like protein containing a CARD
CLRs: C-type lectin receptors
cGAMP: Cyclic GMP–AMP
cGAS: Cyclic GMP–AMP synthase
cGAS-STING: Cyclic GMP–AMP synthase–stimulator of interferon genes
DAMPs: Damage-associated molecular patterns
DIC: Disseminated intravascular coagulation
ER: Endoplasmic reticulum
EVs: Extracellular vesicles
GM-CSF: Granulocyte-macrophage colony-stimulating factor
HDAC: Histone deacetylase
HIF-1α: Hypoxia-inducible factor-1α
HMGB1: High mobility group box 1
HO-1: Heme oxygenase-1
IFNAR: Interferon-α/β receptor
IRFs: Interferon regulatory factors
ISGs: Interferon-stimulated genes
LGP2: Laboratory of genetics and physiology 2
lncRNAs: Long non-coding RNAs
LPS: Lipopolysaccharide
LRR: Leucine-rich repeat
MAVS: Mitochondrial antiviral signaling protein
MDA5: Melanoma differentiation-associated protein 5
MHC class II: Major histocompatibility complex II
MODS: Multiple organ dysfunction syndrome
MSCs: Mesenchymal stem cells
MyD88: Myeloid differentiation primary response protein 88
NETs: Neutrophil extracellular traps
NIK: NF-κb-inducing kinase
NLRs: NOD-like receptors
NLRP3: NOD-, LRR- and pyrin domain-containing protein 3
NOD: Nucleotide-binding oligomerization domain
NQO1: NAD(P)H quinone oxidoreductase 1
Nrf2: Nuclear factor erythroid 2-related factor 2
PAMPs: Pathogen-associated molecular patterns
PPARs: Peroxisome proliferator-activated receptors
PRRs: Pattern recognition receptors
PTMs: Post-translational modifications
RANK: Receptor activator of NF-κB
RIG-I: Retinoic acid-inducible gene I
RLRs: RIG-I-Like receptors
SAE: Sepsis-associated encephalopathy
scATAC-seq: Single-cell ATAC sequencing
SCFAs: Short-chain fatty acids
scRNA-seq: Single-cell RNA sequencing
SNP: Single nucleotide polymorphism
SOCS: Suppressor of cytokine signaling
Soluble TREM-1: Soluble triggering receptor expressed on myeloid cells-1
Syk: Spleen tyrosine kinase
TAK1: Transforming growth factor-β-activated kinase 1
TBK1: TANK-binding kinase 1
TGF-β: Transforming growth factor-β
TLRs: Toll-like receptors
TNF-α: Tumor necrosis factor-α
TRIF: Toll/interleukin-1 receptor-domain-containing adaptor-inducing interferon-β
UPR: Unfolded protein response
UPS: Ubiquitin-proteasome system
